# Isolation and Characterization of Phosphate Solubilizing Bacteria from Paddy Field Soils in Japan

**DOI:** 10.1264/jsme2.ME21085

**Published:** 2022-05-21

**Authors:** Jean Louise Cocson Damo, Maria Daniela Artigas Ramirez, Shin-ichiro Agake, Mannix Pedro, Marilyn Brown, Hitoshi Sekimoto, Tadashi Yokoyama, Soh Sugihara, Shin Okazaki, Naoko Ohkama-Ohtsu

**Affiliations:** 1 United Graduate School of Agriculture, Tokyo University of Agriculture and Technology, Saiwaicho 3–5–8, Fuchu, Tokyo 183–8509, Japan; 2 National Institute of Molecular Biology and Biotechnology, University of the Philippines Los Baños, Los Baños, Laguna 4031, Philippines; 3 Iriomote Station, Tropical Biosphere Research Center, University of the Ryukyus, 870 Uehara, Yaeyama, Taketomi, Okinawa 907–1541, Japan; 4 Faculty of Agriculture, Utsunomiya University, Utsunomiya 321–8505, Japan; 5 Faculty of Food and Agricultural Sciences, Fukushima University, Kanayagawa 1, Fukushima, Fukushima 960–1296, Japan; 6 Institute of Agriculture, Tokyo University of Agriculture and Technology, Saiwaicho 3–5–8, Fuchu, Tokyo 183–8505, Japan; 7 Institute of Global Innovation Research, Tokyo University of Agriculture and Technology, Saiwaicho 3–5–8, Fuchu, Tokyo 183–8509, Japan

**Keywords:** Japan, phosphate solubilizing bacteria, rice, soil phosphorus fractionation

## Abstract

Phosphorus (P) is abundant in soil and is essential for plant growth and development; however, it is easily rendered insoluble in complexes of different types of phosphates, which may lead to P deficiency. Therefore, increases in the amount of P released from phosphate minerals using microbial inoculants is an important aspect of agriculture. The present study used inorganic phosphate solubilizing bacteria (iPSB) in paddy field soils to develop microbial inoculants. Soils planted with rice were collected from different regions of Japan. Soil P was sequentially fractionated using the Hedley method. iPSB were isolated using selective media supplemented with tricalcium phosphate (Ca-P), aluminum phosphate (Al-P), or iron phosphate (Fe-P). Representative isolates were selected based on the P solubilization index and soil sampling site. Identification was performed using 16S rRNA and *rpoB* gene sequencing. Effectiveness was screened based on rice cultivar Koshihikari growth supplemented with Ca-P, Al-P, or Fe-P as the sole P source. Despite the relatively homogenous soil pH of paddy field sources, three sets of iPSB were isolated, suggesting the influence of fertilizer management and soil types. Most isolates were categorized as *β-Proteobacteria* (43%). To the best of our knowledge, this is the first study to describe the genera *Pleomorphomonas*, *Rhodanobacter*, and *Trinickia* as iPSB. *Acidovorax sp.* JC5, *Pseudomonas sp.* JC11, *Burkholderia sp.* JA6 and JA10, *Sphingomonas sp.* JA11, *Mycolicibacterium sp.* JF5, and *Variovorax sp.* JF6 promoted plant growth in rice supplemented with an insoluble P source. The iPSBs obtained may be developed as microbial inoculants for various soil types with different P fixation capacities.

Phosphorus (P) is essential for plant growth and development. Although there is an abundance of P in the soil, only 0.1% of total P (TP) is available for plant uptake (Zou *et al.*, 1992). P is easily rendered insoluble in complexes of different types of phosphates. Stable minerals, such as variscite (AlPO_4_.2H_2_O) and strengite (FePO_4_.2H_2_O), are commonly present in acidic agricultural soils (Richardson, 2001). In addition, soluble orthophosphate is rarely detected in alkaline soils, which contain abundant amounts of calcium (Goldstein *et al.*, 1999). The insoluble nature of P leads to P deficiency and limits crop productivity. Furthermore, there has been a depletion of P reserves (Cordell *et al.*, 2009) because it is a non-renewable source and there is an increasing demand for the bulk production of fertilizers (Dorozhkin, 2011). The production and utilization processes associated with P fertilizers are environmentally undesirable and costly because of the high prices driven by increasing demand, which may place a burden on farmers (Vassilev *et al.*, 2006). Furthermore, the excessive usage of chemical P fertilizers poses a threat to the environment, specifically water pollution (Bennett *et al.*, 2001). An increased dependence on chemical fertilizers and pesticides poses environmental issues, such as air and groundwater pollution, due to the eutrophication of water bodies (Youssef and Eissa, 2014). Therefore, phosphate fertilization is a major agricultural research matter.

An efficient, low-cost, and environmentally friendly approach to soil P fertilization needs to be developed. Specific species of microorganisms with unique properties are often used as alternatives to reduce the use of chemical fertilizers (Deepali and Gangwar, 2010). Phosphate solubilizing microorganisms (PSM) convert insoluble P compounds into soluble forms that are suitable for plant uptake by metabolizing root-borne C compounds, mainly sugars and organic acids (Rodríguez and Fraga, 1999; Whitelaw, 2000; Arcand and Schneider, 2006). Some species of *Pseudomonas*, *Enterobacter*, *Serratia*, *Pantoea*, *Aspergillus*, and *Penicillium* actively solubilize phosphate (Whitelaw, 2000; Son *et al.*, 2006; Buch *et al.*, 2008; Gulati *et al.*, 2008; Sulbarán *et al.*, 2009). These bacteria and fungi grow in media with insoluble phosphate compounds as the sole source of phosphate. PSM not only assimilate P, they also release a large portion of soluble P in excess of their own requirements. The solubilization of mineral phosphates is an important plant growth-promoting (PGP) ability and PSM are now being used as inoculants for crop production.

In Japan, the application of chemical P fertilizers decreased between 1985 and 2005 (Mishima *et al.*, 2010). For example, the application of chemical P fertilizers in paddy fields decreased from 117.0‍ ‍kg P ha^–1^ in 1985 to 83.6‍ ‍kg P ha^–1^ in 2005. Although there was a decline in the utilization of P fertilizers, a reduction in P uptake by paddy rice was not observed. This finding indicated that the application of chemical P fertilizers may be reduced without adverse effects on paddy rice yield and was attributed to most of the P input remaining in farmland soil (Mishima *et al.*, 2003). Applied P is utilized by crops or fixed in complexes. Fixed P may remain in the complex or move to an available pool for crop utilization. Therefore, P for crops comes from P applied in that year or in previous years. Nishio (2003) suggested that the aim in Japan needs to be reductions in P fertilizer input because of the enrichment of soil-available P and the higher levels of P applied than in other countries. Therefore, there is great interest in the solubilization of bound phosphates in soil that have accumulated because of the excessive and repeated use of chemical P fertilizers.

Increases in the levels of P released from phosphate minerals by microbial inoculants are an important aspect of agriculture. The ability of PSM to solubilize bound phosphates and increase the availability of P for rice is a possible mechanism for plant growth promotion under field conditions (Verma *et al.*, 2001). Previous studies reported that the application of PSM, either alone or in combination, to paddy fields enhanced growth and reduced the application of chemical fertilizers. The inoculation of rice grown in Iranian paddy fields with single phosphate solubilizing bacteria (PSB) enhanced rice growth and reduced the application of triple super phosphate by 67% (Bakhshandeh *et al.*, 2015). Furthermore, PSM in combination with other plant growth-promoting rhizobacteria improved agronomic traits, such as root lengths and the biomass of rice grown in organic paddy fields across India (Sherpa *et al.*, 2021). Therefore, PSM may be used as microbial inoculants for rice production.

The present study investigated the potential of inorganic phosphate solubilizing bacteria (iPSB) dwelling in the soil around rice roots for development as microbial inoculants. Soils from paddy rice fields were collected across different regions of Japan. A sequential P extraction ana­lysis was then performed on soil samples. iPSB were isolated using different inorganic P sources. Previous studies on the isolation of PSM only used tricalcium phosphate. In the present study, aluminum phosphate and iron phosphate were also used to isolate iPSB candidates for inoculant development. This approach allowed for the isolation of iPSB and their application to acidic or alkaline soils. To the best of our knowledge, this is the first study to survey iPSB from paddy rice fields across different regions of Japan. The isolates obtained were genetically characterized and their phosphate solubilizing and plant growth-promoting abilities were evaluated.

## Materials and Methods

### Soil sampling and sequential P extraction

**Fig. 1.**
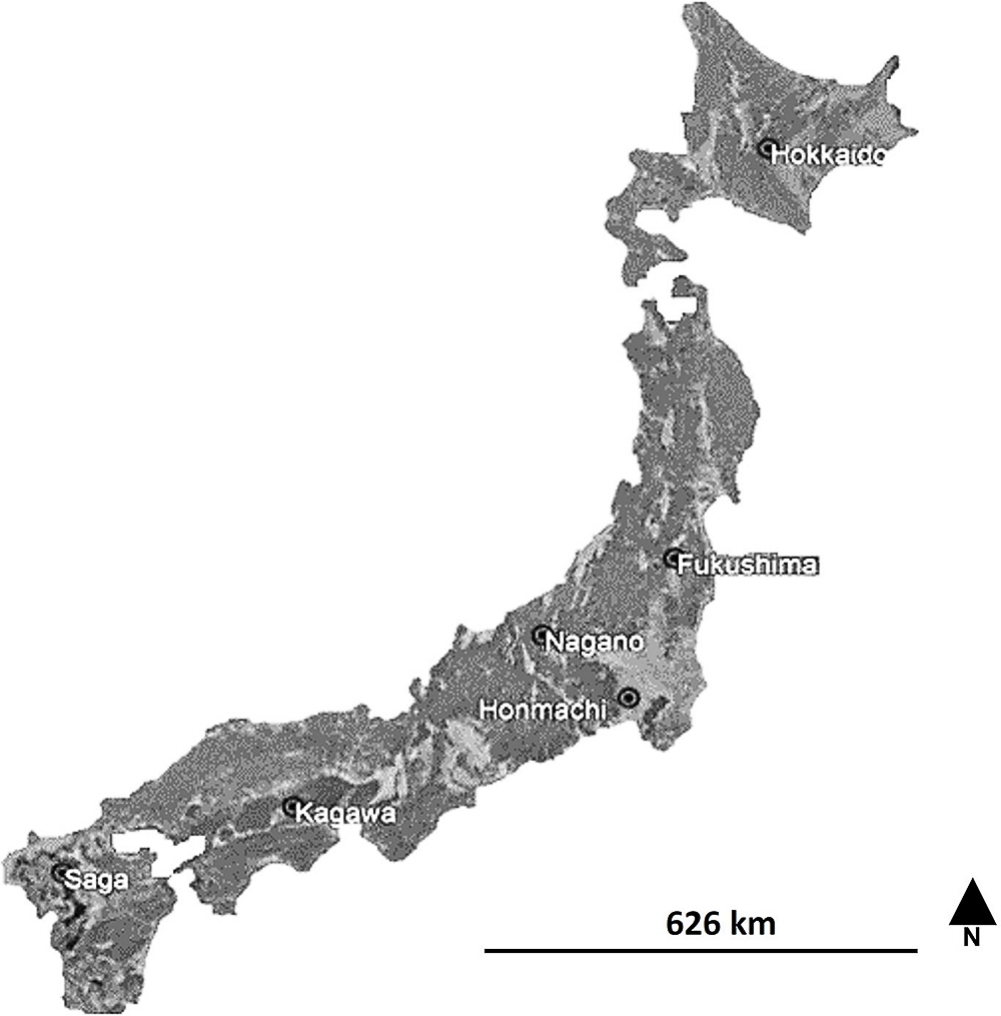
Geographical locations of soil samples used in the isolation of phosphate solubilizing bacteria.

Soils adhering to roots (SAR) were sampled from paddy fields at eight locations in Fukushima, Hokkaido, Honmachi, Kagawa, Nagano, and Saga, Japan (Fig. 1 and Table S1). Soils were sampled at depths of 0–20‍ ‍cm and soil pH was assessed using a 1:5 ratio of soil to deionized water (van Reeuwijk, 2002). The depth of sampling considered the active part for the fibrous roots of rice plants. SAR were collected and stored at 4°C until needed.

Air-dried soils were subjected to HNO_3_-HClO_4_ digestion and TP was quantified using the molybdate blue method (Murphy and Riley, 1962). Soil P was sequentially fractioned using a modified Hedley fractionation protocol (Hedley *et al.*, 1982; Imai *et al.*, 2019). Soil (0.5 g) passed through a 2-mm soil sieve was added to a 50-mL centrifuge tube and sequentially extracted by adding 30‍ ‍mL of each extractant solution. Extractant solutions were added in the following order: distilled water with two resin strips (anion exchange resins in the bicarbonate form), 0.5 M NaHCO_3_ (pH 8.5), 0.1 M NaOH, and 1 M HCl. In all extractions, tubes were shaken for 16 h at 25°C. After each extraction, tubes were centrifuged at 1,339×*g* for 20‍ ‍min. Supernatants were filtered (5B Advantec) and P concentrations were measured using the molybdate blue method after pH adjustments of the solution. Absorbable and loosely bound P was extracted using a resin strip and 0.5 M NaHCO_3_, Al- or Fe-bound P was extracted using 0.1 M NaOH, and Ca-bound P was extracted using 1 M HCl. TP in the NaHCO_3_ and NaOH fractions was digested using (NH_4_)_2_S_2_O_8_ in an autoclave at 120°C for 60 and 90‍ ‍min, respectively. P concentrations were measured using the molybdate blue method. The difference between TP and the sum of all fractionated P was defined as residual P.

### Isolation of iPSB

SAR (10 g) were suspended in 90‍ ‍mL 0.85% NaCl solution using an orbital shaker for 60‍ ‍min. Serially diluted soil suspensions were then plated in National Botanical Research Institute P (NBRIP) medium containing the following (pH 7.0) (g L^–1^): 10 glucose, 0.1 (NH_4_)_2_SO_4_, 0.2 KCl, 0.25 MgSO_4_.7H_2_O, 5 MgCl_2_.6 H_2_O, 1.8% agar, and 5 Ca_3_(PO_4_)_2_ (Ca-P) as the sole insoluble P source. Modified Reyes medium (Gadagi and Sa, 2002) contained (pH 6.5) (g L^–1^) 0.1 NaCl, 0.5 MgSO_4_.7H_2_O, 0.1 CaCl_2_.2H_2_O, 0.0005 FeSO_4_.7H_2_O, 0.00156 MnSO_4_.H_2_O, 0.00140 ZnSO_4_.7H_2_O, 2‍ ‍μg vitamin B12, 30 sucrose, 1.8% agar, 0.5% bromocresol green, and 5 AlPO_4_ (Al-P) or FePO_4_ (Fe-P) as the sole insoluble P source. Culture plates were incubated at 28°C for 3–7 days. Colonies that formed halo zones were considered to be iPSB (Nautiyal, 1999). Colonies having halo zones and showing different colony morphologies at a 1/1,000 dilution were selected as isolates. Single colonies were selected, re-cultured in tryptic soy (TS) medium, NBRIP for Ca-P isolates, or Reyes’ medium for Al-P and Fe-P isolates, and maintained as 40% glycerol stocks at –80°C until needed.

### Estimation of phosphate solubilizing ability

A total of 147 bacterial isolates were grown in TS broth at 28°C for 24‍ ‍h. Bacterial cells were centrifuged at 10,000×*g* for 15‍ ‍min. The supernatant was discarded and cells were resuspended in 0.85% NaCl solution at a cell density of 10^7^ colony forming units mL^–1^ (CFU mL^–1^). Resuspended cells (10‍ ‍μL) were transferred to medium plates containing NBRIP supplemented with 5‍ ‍g L^–1^ Ca_3_(PO4)_2_ for Ca-P isolates, Reyes medium with AlPO_4_ for Al-P isolates, or Reyes medium amended with FePO_4_ for Fe-P isolates. There were three replicates of each isolate type. Plates were incubated at 30°C for 7 days. The formation of a halo zone around the bacterial colony indicated that the isolate solubilized phosphate. The phosphate solubilizing ability of each isolate was evaluated by measuring the size of the halo zone, and the phosphate solubilization (PS) index was calculated as previously described (Premono Edi *et al.*, 1996). Isolates that showed high PS indices were selected for genetic characterization and plant assays.

A second screening was performed to quantitatively estimate the phosphate solubilizing ability of isolates. Representative isolates with high PS indices were grown in TS broth at 28°C for 24‍ ‍h and cell density was adjusted to 10^8^ CFU mL^–1^. The bacterial suspension was then transferred to P growth media supplemented with Ca_3_(PO4)_2_, AlPO_4_, or FePO_4_. After a 5-day incubation at 28°C under shaking at 130‍ ‍rpm, the supernatant was collected by centrifugation at 13,000×*g* for 10‍ ‍min. Soluble P released in the supernatant was quantified using the molybdenum blue assay (Murphy and Riley, 1962). The experiment was performed in triplicate. Uninoculated samples served as negative controls. The positive control bacterium used was *Priestia* (*Bacillus*) *megaterium* NBRC 15308, which is a known phosphate solubilizer (Rodríguez and Fraga, 1999), and was supplied by the National Institute of Technology and Evaluation (NITE) Biological Resource Center.

### Extraction of genomic DNA

Isolates were grown in TS broth at 28°C for 24 h. Cells were collected by centrifugation at 13,000×*g* for 2‍ ‍min and washed with sterile distilled water. Genomic DNA was extracted using a Wizard^®^ Genomic DNA Purification Kit (Promega) according to the manufacturer’s protocol.

### DNA amplification and sequencing

The 16S rRNA and *rpoB* regions of 63 out of the 147 isolates were sequenced. A polymerase chain reaction (PCR) and sequencing of the 16S rRNA gene were performed using the bacterial universal primers 1F (5′-AGT TTG ATC CTG GCT C-3′) and 3R (5′-AAG GAG GTG ATC CAG CC-3′), (Habibi *et al.*, 2014), and rpoB-F (5′-ATCGAAACGCCTGAAGGTCCAAACAT-3′) and rpoB-R (5′-ACACCCTTGTTACCGTGACGACC-3′) for the *rpoB* gene (Mohkam *et al.*, 2016). Genes were amplified using 10‍ ‍ng of purified DNA and KOD Plus Neo (Toyobo). PCR products were purified using a FastGene™ Gel/PCR Extraction Kit (Nippon Genetics) and then sequenced using an ABI PRISM 3500 Genetic Analyzer (Applied Biosystems) according to the manufacturer’s instructions. The sequences obtained were compared to the 16S rRNA and *rpoB* gene sequences deposited in the GenBank database using BLAST online software (http://www.ncbi.nlm.nih.gov/BLAST). Phylogenetic trees based on the nucleotide sequences of the 16S rRNA and *rpoB* gene sequences were aligned using Genetyx version 11 (Genetics). Phylogenetic trees were constructed based on the neighbor-joining algorithm with 1,000 replications using the bootstrap method and the maximum composite likelihood model without topology. These processes were conducted using Molecular Evolutionary Genetics Analysis (MEGA) software (version 6.0; Pennsylvania State University, State College, PA, USA) (Tamura *et al.*, 2013).

### Nucleotide sequence accession numbers

DNA sequences were deposited in the DNA Data Bank of Japan (DDBJ) under accession numbers LC661699 to LC661761 (16S rRNA) and LC661762 to LC661819 (the *rpoB* gene).

### Plant growth assay

Sixty-three representative isolates were cultured in TS broth at 28°C for 24 h with constant shaking and bacterial cells were then collected by centrifugation and resuspended in 0.85% NaCl solution. *Oryza sativa* L. ‘Koshihikari’ (rice) seeds were surface-sterilized by soaking in 70% ethanol for 30‍ ‍s followed by 3% sodium hypochlorite for 3‍ ‍min. They were then washed five times with sterile distilled water. The sole insoluble P source of 85‍ ‍mg of Ca_3_(PO_4_)_2_ for Ca-P isolates, AlPO_4_ for Al-P isolates, and FePO_4_ for Fe-P isolates was amended in 1‍ ‍kg of sterilized vermiculite (Subero *et al.*, 2016). Sterilized seeds were incubated at 28°C in the dark for 48 h before germination. Pre-germinated seeds were transplanted into tubes (outer diameter: 29.1‍ ‍mm×length: 114.4‍ ‍mm) with 20‍ ‍g vermiculite (Vermitech), and seeds were then inoculated with 200‍ ‍μL of the bacterial culture at 10^8^ CFU mL^–1^. Plants were grown in a growth chamber room under controlled environmental conditions with 16 h light (5,000–7,000 lx) and 8 h darkness, and were maintained at 28±2°C. Each container was irrigated with sterile distilled water at a 100% water-holding capacity for vermiculite throughout the cultivation period. The experiment was a completely randomized design with three replicates for each treatment, and each treatment contained six plants. Uninoculated plants and plants inoculated with *Pseudomonas veronii* JR37 were used as negative and positive controls, respectively (Habibi *et al.*, 2014). Plants were harvested after 2‍ ‍weeks and the roots were washed with tap water to remove adhering vermiculite. Fresh shoot and root lengths were measured. Shoots and roots were then dried at 80°C for 48 h before measuring their dry weights. Comparisons between the control and treatment groups were performed using a one-way ANOVA and Dunnett’s post hoc ana­lysis at *P*<0.05 by SAS software (SAS Institute).

## Results

### Soil P ana­lysis

Sequential P extraction was performed on eight rice soil samples. The TP of soil samples ranged between 6,076 and 21,638‍ ‍mg P kg^–1^. Table S2 shows that SAR from Honmachi had the highest TP among the soil samples examined, while SAR from Fukushima site 2 had the lowest.

**Fig. 2.**
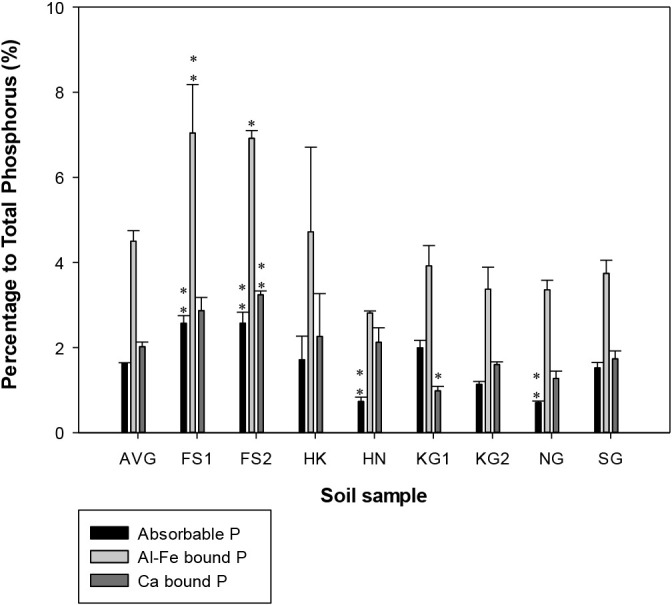
Percentage of fractionated phosphorus to total phosphorus in soil samples. AVG, average value of soil samples; FS1, Fukushima site 1; FS2, Fukushima site 2; HK, Hokkaido; HN, Honmachi; KG1, Kagawa site 1; KG2, Kagawa site 2; NG, Nagano; and SG, Saga. The significance of differences between the overall average and soil samples was assessed by Dunnett’s test (*** *P*<0.001, ** *P*<0.01, * *P*<0.05). Error bars indicate the standard deviation of three replicates.

The percentages of different P fraction contents in each fraction to TP are shown in Fig. 2. Absorbable P accounted for only 1.6% of TP on average, and both sites in Fukushima had the highest absorbable P percentages (2.6%), whereas Nagano (0.7%) had the lowest. The two SAR samples from Fukushima had significantly different absorbable P values from the average for all soil samples. In contrast, SAR from Nagano and Honmachi had significantly lower absorbable P values. Al- or Fe-bound P ranged between 295 and 750‍ ‍mg P kg^–1^, corresponding to between 7.0 and 2.8% TP. Both SAR from Fukushima had significantly higher percentages of Al-Fe bound P than the average for all soil samples. In addition, Ca-bound P at Fukushima site 2 had the highest Ca-bound P percentage to TP (3.2%), while Kagawa site 1 had the lowest (1.0%). Residual P comprised 92% TP averaged over all soil samples, ranging between 87.3% (Fukushima site 1) and 94.7% (Nagano). In contrast to absorbable P, both samples from Fukushima had significantly lower residual P values than the average for all samples.

### Isolation of iPSB

Eight paddy rice SAR were used to isolate the three types of iPSB. The number of phenotypically different isolates obtained per SAR was noted; 147 isolates were obtained (Table 1). Sixty isolates solubilized tricalcium phosphate (Ca-iPSB), 30 solubilized aluminum phosphate (Al-iPSB), and 57 solubilized iron phosphate (Fe-iPSB). The largest numbers of iPSB were isolated from Kagawa site 2, which was an Entisol and organically fertilized. Among all isolates, the highest percentages of Ca-iPSB were recorded for Fukushima site 1 and Kagawa site 2. Fukushima site 1 was classified as an Andisol and was managed using chemical fertilizers. The highest ratio of Al-iPSB was recorded at Kagawa site 1, whereas Fe-iPSB were isolated from Nagano. Soil samples from both sites were categorized as Entisols with organic fertilizer management.

The relationship between the P content in each fraction of soil samples and isolated iPSB was analyzed (Table 2). The Ca-bound P content negatively correlated (r=–0.74) with the number of isolated Al-iPSB. A negative correlation (r=–‍0.75) was also observed between the number of isolated Ca-iPSB and Fe-iPSB. In addition, a positive correlation (r=0.91) was noted between absorbable P and Al-Fe-bound P, while a negative correlation (r=–0.91) was found with the residual soil P content. Negative correlations were also observed between the residual P content and Al-Fe bound P (r=–0.99) and Ca-bound P (r=–0.86). Al-Fe-bound P positively correlated with Ca-bound P (r=0.81).

### Estimation of phosphate solubilizing ability

PS indices were measured for 147 iPSB. Thirty out of 60 Ca-iPSB with a high phosphate solubilizing ability (Fig. S1A and Table 1) were selected for genetic characterization and plant assays. JC1 isolated from Fukushima site 1 had the highest PS index (2.90) among the representative isolates, whereas JC24 (1.28) from Saga had the lowest. Furthermore, 16 representative isolates with a high phosphate solubilizing ability were selected from 30 Al-iPSB samples (Fig. S1B and Table 1). JA6 (6.98) from Kagawa site 1 had the highest PS index out of the Al-iPSB isolates. Seventeen out of the 57 Fe-iPSB isolates showed a high phosphate solubilizing ability and were selected for further ana­lyses. JF17 had the maximum PS index in the iron phosphate group (Fig. S1C and Table 1).

**Fig. 3.**
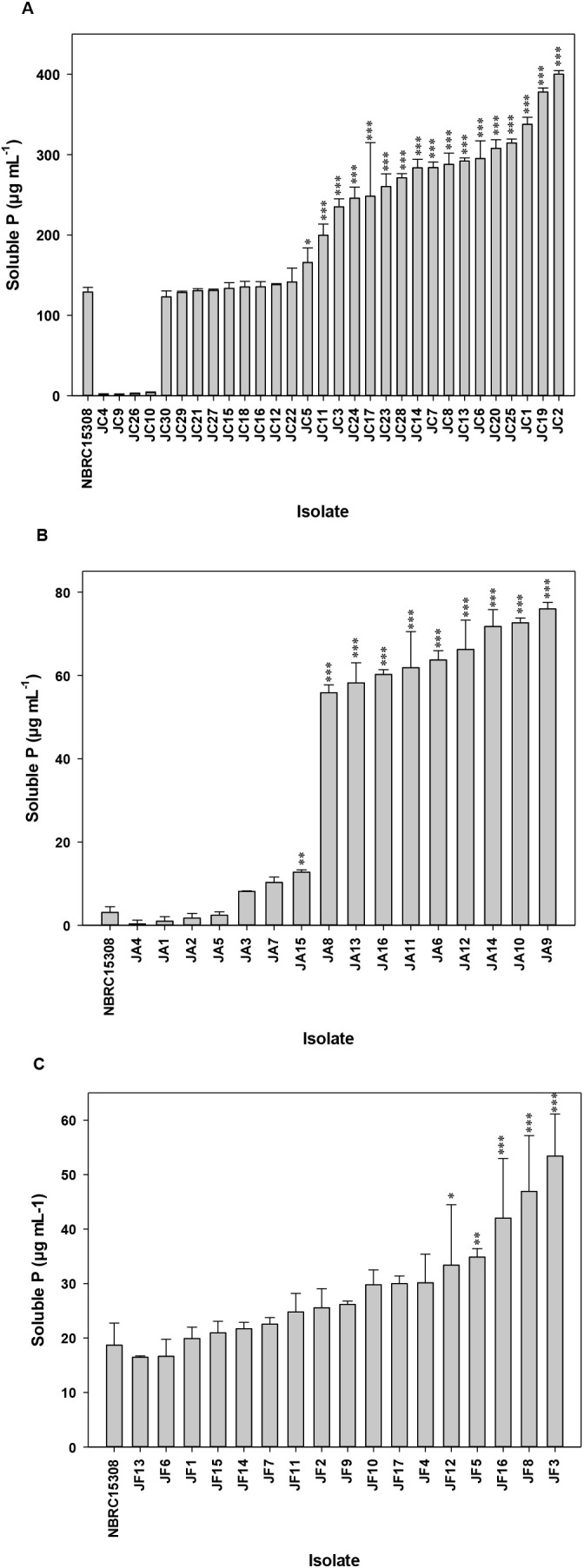
Quantitative estimation of phosphate solubilization by representative isolates on P growth media amended with tricalcium phosphate (A), aluminum phosphate (B), or iron phosphate (C). Values represent the net soluble P by deducting the value of uninoculated samples. NBRC 15308 served as the positive control. The significance of differences between NBRC 15308 and isolates was assessed by Dunnett’s test (*** *P*<0.001, ** *P*<0.01, * *P*<0.05). Means and standard deviations (*n*=3) are shown.

To further confirm the phosphate solubilizing ability of the representative isolates, the quantification of released P from insoluble P sources was performed. Seventeen isolates exhibited significantly higher solubilized P from Ca_3_(PO_4_)_2_ than the positive control (Fig. 3A). JC2 from Fukushima site 1 had the maximum PS (400‍ ‍μg mL^–1^) among the representative isolates. In addition, 10 isolates exhibited the significant release of soluble P from AlPO_4_ over NBRC 15308 (Fig. 3B). JA9 obtained from Kagawa site 1 had the highest amount of soluble P (76‍ ‍μg mL^–1^) out of the Al-iPSB isolates. Five isolates had a larger amount of P solubilized from FePO_4_ than the positive control (Fig. 3C). Isolate JF3 from Fukushima site 1 had the highest amount of soluble P (53‍ ‍μg mL^–1^) for the Fe-iPSB group.

### Genetic characterization based on 16S rRNA and rpoB sequencing

**Fig. 4.**
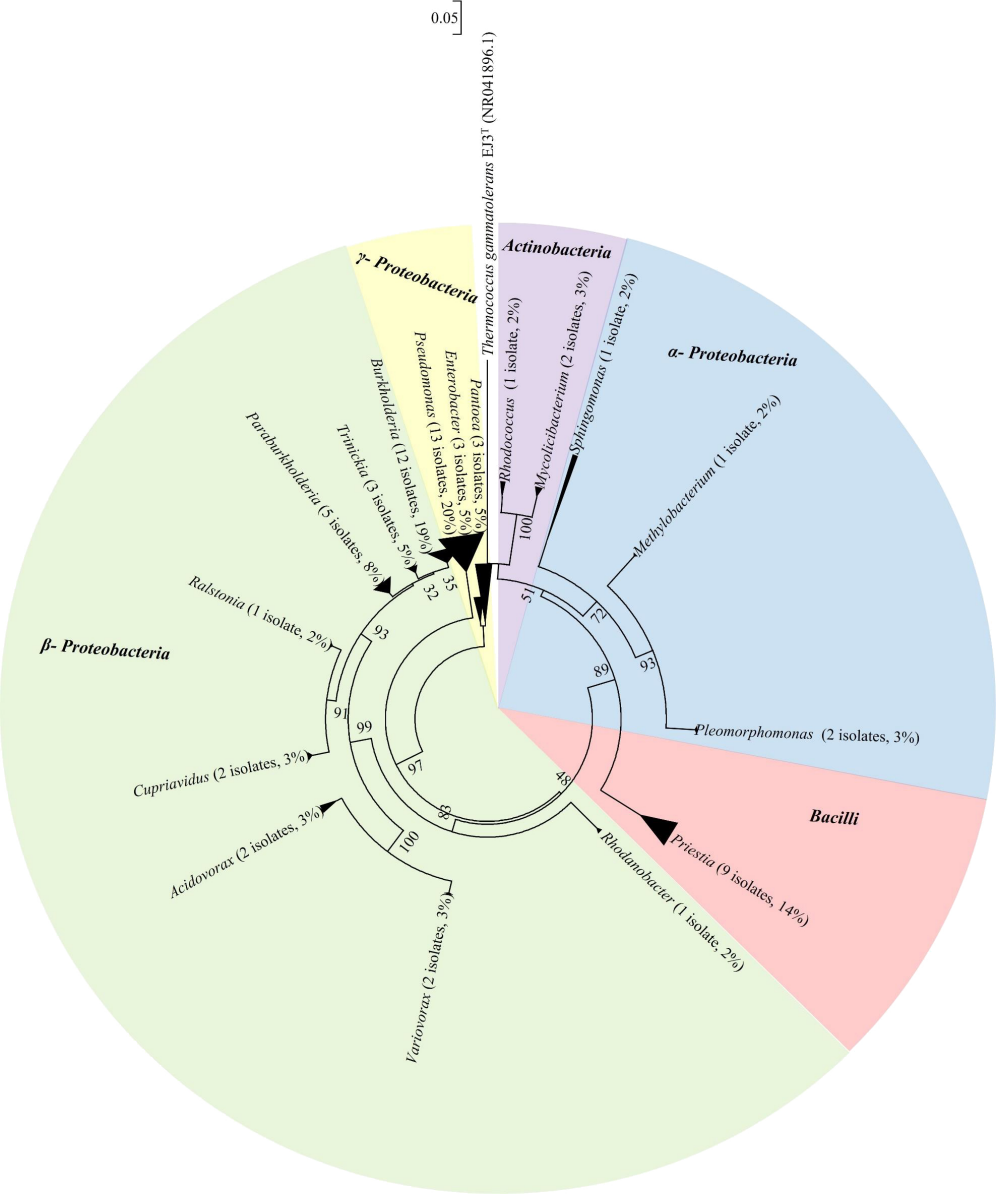
Composition of representative phosphate solubilizing bacteria based on 16S rRNA gene sequencing. Numbers at nodes indicate the level of bootstrap support (%) based on the 1,500-bp DNA fragment and neighbor-joining ana­lysis with 1,000 replications. The scale bar indicates 0.05 changes per site.

Representative isolates were subjected to 16S rRNA and *rpoB* sequence ana­lyses. Sixty-three isolates were selected based on the SAR source and their phosphate solubilizing ability (Table 1 and Fig. S1). The closest reference strains (with >98% similarity) for each isolate were summarized in the phylogenetic ana­lysis (Fig. 4) and their phenotypes with PS indices are shown in Table 3.

Phylogenetic relationships among the 63 iPSB isolates were strengthened using an outgroup (*Thermococcus gammatolerans* EJ3), as shown in Fig. S2. The phylogenetic ana­lysis classified the representative isolates into the following groups: *Actinobacteria* (5%), *Bacilli* (14%), *α-Proteobacteria* (6%), *β-Proteobacteria* (43%), and *γ-Proteobacteria* (32%) (Fig. 4). The majority of iPSB were categorized as *β-Proteobacteria*, with *Burkholderiaceae* as the dominant family in the present study. The *Actinobacteria* group was composed of *Rhodococcus* (one isolate) and *Mycolicibacterium* (two isolates). *Priestia* isolates (nine) were *Bacilli*. The *α-Proteobacteria* group had one isolate closely related to *Methylobacterium*, one *Sphingomonas*, and two *Pleomorphomonas*. The *γ-Proteobacteria* group contained 20 isolates with reference strains for *Pseudomonas* (13 isolates), *Rhodanobacter* (1 isolate), *Enterobacter* (3 isolates), and *Pantoea* (3 isolates). Most of the isolates were categorized as *β-Proteobacteria*. This group contained two *Acidovorax* iPSB isolates, two *Variovorax* isolates, two *Cupriavidus* isolates, one *Ralstonia* isolate, five *Paraburkholderia* isolates, three *Trinickia* isolates, and 12 *Burkholderia* isolates.

One housekeeping gene (*rpoB*) was phylogenetically analyzed to confirm the identity of iPSB (Fig. S3). In general, the results obtained were consistent with 16S rRNA results. Most of the isolated iPSB were *β-Proteobacteria*. However, the *rpoB* gene was not successfully amplified from *Actinobacteria* or the genus *Pleomorphomonas*. To the best of our knowledge, the genera *Pleomorphomonas* (JA1 and JA2), *Rhodanobacter* (JF8), and *Trinickia* (JC30, JF12, and JF16) were described herein for the first time as phosphate solubilizing bacteria.

### Soil characteristics and iPSB genera

**Fig. 5.**
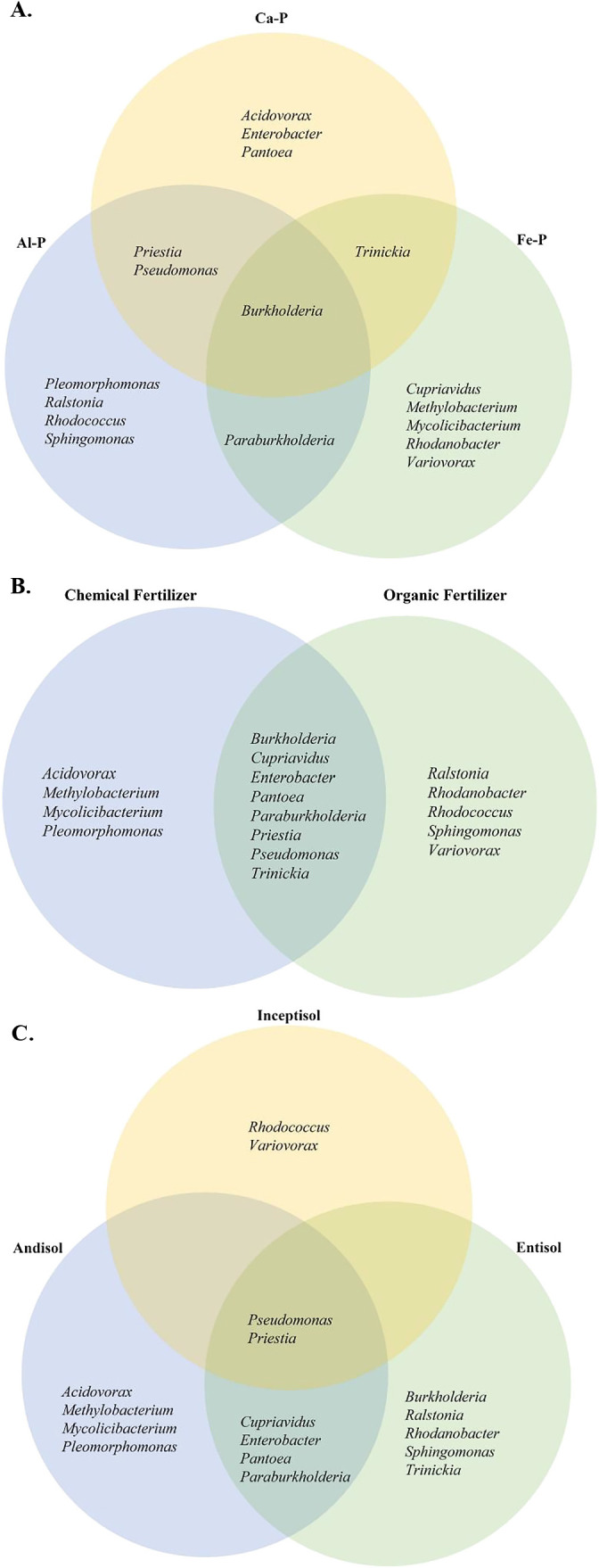
Venn diagram for isolated iPSB genera Unique and/or shared genera among the types of inorganic P used to isolate bacteria (A), between fertilizer management regimes (B), and soil types (C) in paddy field sources. Al-P: AlPO_4_, Ca-P: Ca_3_(PO_4_)_2_, Fe-P: FePO_4_

Comparisons of isolated iPSB genera were based on the type of inorganic P source used in isolation, fertilizer management, and the soil type of SAR sources (Fig. 5). *Acidovorax*, *Enterobacter*, and *Pantoea* were obtained after Ca-P isolation, whereas *Pleomorphomonas*, *Ralstonia*, *Rhodococcus*, and *Sphingomonas* were uniquely isolated using Al-P (Fig. 5A). In addition, Fe-P isolation yielded *Cupriavidus*, *Methylobacterium*, *Mycolicibacterium*, *Rhodanobacter*, and *Variovorax*. A few bacterial genera were also shared by two types of inorganic P sources, such as *Trinickia* from Ca-P and Fe-P isolation, *Pseudomonas* and *Priestia* from Ca-P and Al-P isolation, and *Paraburkholderia* from Al-P and Fe-P isolation. *Burkholderia* was isolated from inorganic P sources.

The bacterial genera *Burkholderia*, *Cupriavidus*, *Enterobacter*, *Pantoea*, *Paraburkholderia*, *Priestia*, *Pseudomonas*, and *Trinickia* were isolated regardless of the fertilizer management applied to the SAR source (Fig. 5B). In contrast, *Acidovorax*, *Methylobacterium*, *Pleomorphomonas*, and *Mycolicibacterium* were only isolated in paddy fields in which chemical fertilizers had been used, whereas *Ralstonia*, *Rhodanobacter*, *Rhodococcus*, *Sphingomonas*, and *Variovorax* were exclusively obtained from paddy fields managed with organic fertilizer.

The soil type of the Rh source was also considered to clarify whether it had any effects on the type of iPSB isolated (Fig. 5C). *Rhodococcus* and *Variovorax* were isolated from Inceptisols, whereas *Acidovorax*, *Methylobacterium*, *Mycolicibacterium*, and *Pleomorphomonas* were found in Andisols. *Burkholderia*, *Ralstonia*, *Rhodanobacter*, *Sphingomonas*, and *Trinickia* were isolated from Entisols, and *Cupriavidus*, *Enterobacter*, *Pantoea*, and *Paraburkholderia* were found in Andisol and Entisol soils. *Pseudomonas* and *Priestia* were obtained from all soil types.

### Effects of bacterial inoculation on rice plant growth promotion

To evaluate the effects of iPSB on rice plant growth, representative isolates (63) were inoculated onto *O. sativa* Koshihikari planted in sterilized vermiculite amended with insoluble P. *P. veronii* JR37 (AB793766; Habibi *et al.*, 2014) was also included in the plant assay as a positive control.

A summary of rice plant growth promotion (PGP) by iPSB is shown in Table 4. Regardless of the insoluble P used, some isolated iPSB performed better than the positive control *P. veronii* JR37. In the Ca-iPSB plant assay using Ca_3_(PO_4_)_2_ as the sole P source, the maximum dry biomass (shoot, root, and total) was recorded for rice plants inoculated with *Acidovorax sp.* JC1 (Fig. S4). In comparisons with plants that had not been inoculated with Ca-P (UN+Ca-P), root lengths were significantly longer in rice plants inoculated with *Acidovorax sp.* JC5 and *Pseudomonas sp.* JC11. These isolates were isolated from Fukushima: *Acidovorax* from Site 1 and *Pseudomonas* from Site 2.

The results of the AlPO_4_ assay showed that rice plants inoculated with *Burkholderia sp.* JA6 from Kagawa site 1 and *Sphingomonas sp.* JA11 from Nagano had the longest root lengths (Fig. S5). In addition, rice plants inoculated with the Kagawa site 2 isolate *Burkholderia sp.* JA10 has significantly longer root lengths than those that had not been inoculated with Al-P (UN+Al-P). Among the Al-iPSB used, *Burkholderia sp.* JA9 from Kagawa site 1 produced the highest shoot and total dry weights. Rice plants inoculated with *Ralstonia pickettii* JA12 from Nagano had the highest root dry weight.

The effects of Fe-iPSB on rice were examined using FePO_4_ as an insoluble P source. Shoot lengths were affected by six isolates from 17 representative Fe-iPSBs (Fig. S6). *Mycolicibacterium sp.* JF3 had the longest shoot length among Fe-iPSB. Moreover, 16 isolates had longer root lengths than the insoluble P-amended control. Rice plants inoculated with *Variovorax sp.* JF6 (Hokkaido) and *Mycolicibacterium sp.* JF5 (Honmachi) had significantly longer root lengths than UN+Fe-P. Rice plants inoculated with *Rhodanobacter sp.* JF8 from Nagano had the highest shoot dry weights, whereas *Burkholderia sp.* JF14 from Kagawa site 1 had the highest root dry weight. Rice plants inoculated with *Burkholderia sp.* JF15 (Kagawa site 2) had the highest total dry weight.

The majority of promising iPSB were isolated from paddy fields with organic fertilizer management (Table 4). Additionally, the top isolates showed longer root lengths of the rice cultivar Koshihikari with insoluble P as the sole P source than the control.

## Discussion

### Soil P content and iPSB

The soil P content and its forms were evaluated in the SAR samples used to isolate the three sets of iPSB (Fig. 2 and Table S2). P bioavailability and stability were previously shown to significantly depend on soil characteristics (Wright, 2009). Among the soil samples examined, SAR from Honmachi had the highest TP content. It was an Andisol, which generally has a high P fixation capacity. However, SAR from Fukushima site 1 was also an Andisol, but had a low TP content. SAR from Saga, classified as an Entisol, had the lowest TP content. Entisols have inherently low TP and a poor P fixation capacity (Dissanayaka *et al.*, 2015). Furthermore, the P forms that had accumulated in croplands in which P was in predominantly inorganic forms were affected by the application of fertilizers and manure (Ando *et al.*, 2021). Ca-bound P at Fukushima site 2 was present at the highest percentage among TP in all samples. In this field, chemical fertilizers were applied during the rice-planting season. The lowest percentage of Ca-bound P was observed at Kagawa site 1, which was an organically cultivated site. This is consistent with the findings by Wright (2009) who reported that the application of superphosphate (a chemical fertilizer) increased the Ca-P content in the surface soils of agricultural land. The present results also revealed a positive correlation between the absorbable P content with Al-P and Fe-P in soil samples, as detected in SAR from Fukushima, which had the largest absorbable P, Al-P, and Fe-P contents. This is in agreement with previous findings showing that labile and relatively stable P forms were influenced by Al and Fe in Japanese soils (Tani *et al.*, 2010; Nakamura *et al.*, 2019).

iPSB were isolated from paddy rice SAR and may be used to develop microbial inoculants. A selective medium without available soluble P sources was used to isolate potential iPSB. Soil pH affects the P forms present, which are mainly Ca-bound P in alkaline soils and Al or Fe complexes in acidic soils. Therefore, three insoluble P sources (Ca_3_[PO_4_]_2,_ AlPO_4_, and FePO_4_) were used to isolate iPSB, yielding three sets of iPSB. Interestingly, there was no universal metal-P compound for the selection of PSM (Bashan *et al.*, 2013). Previous studies reported that the isolation of iPSB using Ca_3_(PO_4_)_2_ alone yielded iPSB with a low to no solubilizing ability with AlPO_4_ and FePO_4_ (Pérez *et al.*, 2007; Park *et al.*, 2010). These findings imply that isolated iPSB are impractical for acidic soils.

Environmental factors generally influence the distribution of bacterial communities. Therefore, the relationship between the soil P content and number of isolated iPSBs was examined. Despite the relatively homogenous soil pH, different types of iPSB, such as those associated with inorganic P (Ca-P, Al-P, and Fe-P), were isolated. Therefore, the types and number of iPSB may be affected by the fertilizer management regime and the soil type of paddy field sources. Among all isolates, the majority of iPSB were obtained from Entisol soils. Although this soil has a low Al/Fe oxide content (Dissanayaka *et al.*, 2015), a large portion of Al-iPSB and Fe-iPSB were isolated from SAR in this soil type. With the exception of Fukushima site 1, a high percentage of Ca-iPSB was taken from Kagawa site 2 with low Ca-bound P. Specific iPSB are more likely to be isolated based on the low content of inorganic P content in soil, such as the high percentage of Al-iPSB originating from soil with low Al-P and Fe-P contents. The discrepancy of high Ca-iPSB in high Ca-bound P soil in Fukushima may be explained by the chemically fertilized nature of the field. The fertilizer management of paddy rice SAR samples must have affected the occurrence of iPSB. Previous studies demonstrated that the application of chemical fertilizers may reduce the abundance of beneficial taxa for plant health and growth (Sinong *et al.*, 2021). This was reflected in the present results, with the highest portion of total iPSB being obtained at Kagawa sites 1 and 2 and at Nagano, at which organic farming is practiced.

### Characterization of iPSB

A total of 147 iPSBs were screened based on their PS indices. Sixty-three isolates were selected in consideration of the SAR from which they were obtained. The results of 16S rRNA and *rpoB* gene sequencing led to iPSB being classified into five groups (Fig. 4, S2 and S3).

The first group was *Actinobacteria*, which was composed of *Rhodococcus* and *Mycolicibacterium*. *Rhodococcus sp.* JA4 from Hokkaido solubilized AlPO_4_, whereas the isolates *Mycolicibacterium sp.* JF3 and JF5 solubilized FePO_4_. The genus *Rhodococcus* possesses plant growth-promoting properties and is commonly isolated in soils polluted with heavy metals (Belimov *et al.*, 2001) and petroleum hydrocarbons (Pacwa-Płociniczak *et al.*, 2016). Although *R. erythropolis* has been shown to solubilize Ca_3_(PO_4_)_2_, its ability to solubilize AlPO_4_ has not yet been demonstrated. The phytoremediation abilities shown by *Rhodococcus* have been examined (Toussaint *et al.*, 2012; Álvarez-López *et al.*, 2016). Since this genus degrades persistent pollutants, isolated *Rhodococcus* exhibits tolerance to heavy metals, such as aluminum. *Mycolicibacterium* may also be obtained from soil contaminated with polycyclic aromatic hydrocarbons (Hormisch *et al.*, 2004). Although *M. fluoranthenivorans* has been applied to food and feed processing, specifically to the degradation of aflatoxin B1 (Hormisch *et al.*, 2004; Teniola *et al.*, 2005), its potential as a PGP bacterium was described herein for the first time.

*Priestia* (formerly known as *Bacillus*) belongs to the *Bacilli* group. The majority of isolated *Priestia* species were Ca-iPSB; however, there was one Al-iPSB. This genus is considered to be a strong solubilizer of tricalcium phosphate (Rodríguez and Fraga, 1999; Li *et al.*, 2019). Previous studies showed that members of the genus exhibited the ability to solubilize P from aluminum phosphate (Banik and Dey, 1983). *Methylobacterium*, *Pleomorphomonas*, and *Sphingomonas* are known plant growth-promoting bacteria and comprise the *α-Proteobacteria* group of isolated iPSB. The plant growth-promoting mechanism includes the production of 1-aminocyclopropane 1-carboxylate deaminase in *M. fujisawaense* and *M. oryzae* (Madhaiyan *et al.*, 2006, 2007); however, the phosphate solubilizing ability of *M. fujisawaense* has not yet been reported. The genus *Pleomorphomonas* was herein identified for the first time as a phosphate solubilizing bacteria. This genus is generally associated with nitrogen-fixing bacteria, such as *Pleomorphomonas oryzae*, which has also been isolated from paddy rice fields (Xie and Yokota, 2005).

The *γ-Proteobacteria* group consisted of *Pseudomonas*, *Enterobacter*, *Pantoea*, and *Rhodanobacter*. Some of the isolated *Pseudomonas* had the capacity to solubilize P from aluminum phosphate. Isolated *Enterobacter* and *Pantoea* were exclusively obtained using tricalcium phosphate, a common inorganic P source for the isolation of iPSB. These genera are typically referred to as phosphate solubilizers. However, it is important to note that the present results showed that *Rhodanobacter* functioned as an iron phosphate solubilizer. The type strain of this bacterium was isolated from a contaminated aquifer with a high nitrate content and was capable of complete denitrification (Prakash *et al.*, 2012).

The majority of iPSB were classified under *β-Proteobacteria* as *Acidovorax*, *Burkholderia*, *Cupriavidus*, *Paraburkholderia*, *Ralstonia*, *Trinickia*, and *Variovorax*. Most of these genera are common phosphate solubilizers, except for Fe-iPSB, which were similar to *Variovorax ginsengisoli*, reported herein for the first time. *V. ginsengisoli* is generally considered to be a denitrifying bacterium that is isolated from ginseng (Im *et al.*, 2010). In addition, the genus *Trinickia* was shown to function as a phosphate solubilizer in this study. This genus is characterized by plant growth-promoting (Fu *et al.*, 2019) and heavy metal-tolerant bacteria (Zhu *et al.*, 2012).

### Plant growth promotion of iPSB

Promising iPSB demonstrated their potential for plant growth promotion in rice with insoluble P as the sole P source. In consideration of the phosphate solubilizing ability of these isolates, positive correlations were observed with PS indices, P solubilization, and plant growth promotion in rice. A positive correlation was also noted between PS indices and plant growth traits in Ca-iPSB, specifically shoot dry weight (r=0.38) and total dry weight (r=0.41). Moreover, positive correlations were found between root length and the PS index (r=0.57) and also P solubilization (r=0.80) in Al-iPSB. Therefore, isolation and screening methods based on specific inorganic phosphates were reliable for Ca-iPSB and Al-iPSB as candidate microbial inoculants. However, no significant relationships were observed for Fe-iPSB. This may be attributed to the isolation method, which heavily depended on the production of acids by bacteria. Another mechanism for the liberation of iron in FePO_4_ may be through siderophores. Therefore, further studies are needed to confirm the relationship between the PGP and phosphate solubilizing abilities of isolates.

The isolates *Acidovorax sp.* JC5 and *Pseudomonas sp.* JC11 increased the root lengths of rice treated with Ca_3_(PO_4_)_2_ and showed the high release of soluble P *in vitro*. Microbial inoculants may be developed using these isolates for soils that are heavily fertilized with chemical P fertilizers because the dominant P species in these soils may be Ca-P. Although available P is high and sufficient for crops in these paddy soils, a portion of the soluble chemical P fertilizer applied will still be rendered insoluble or leach into bodies of water, potentially causing eutrophication. The aim of microbial inoculants with Ca-iPSB under these conditions is to efficiently utilize and ultimately reduce the amount of the chemical P fertilizer used. Moreover, existing bound P from previous cropping seasons may be utilized again by plants through solubilization. The Al-iPSB isolates *Burkholderia sp.* JA6 and JA10 and *Sphingomonas sp.* JA11 improved root lengths and a quantitative estimation revealed higher soluble P than a known P solubilizer by utilizing AlPO_4_. These isolates may be used to develop microbial inoculants for volcanic soils (Andisol), which have a high P fixation capacity due to Al oxides. Root lengths were increased by Fe-iPSB *Variovorax sp.* JF6 and *Mycolicibacterium sp.* JF5 with FePO_4_ as the only P source. The abovementioned Fe-iPSB may potentially be developed as inoculants for red-yellow soils (Inceptisol or Ultisol) with a strong P fixation ability because they contain Fe oxide. However, further studies on field conditions are needed to confirm the potential of these isolates as microbial inoculants for high P fixation areas.

## Citation

Damo, J. L. C., Ramirez, M. D. A., Agake, S., Pedro, M., Brown, M., Sekimoto, H., et al. (2022) Isolation and Characterization of Phosphate Solubilizing Bacteria from Paddy Field Soils in Japan. *Microbes Environ ***37**: ME21085.

https://doi.org/10.1264/jsme2.ME21085

## Supplementary Material

Supplementary Material

## Figures and Tables

**Table 1.** General characteristics of soil samples and the number of inorganic phosphate solubilizing isolates.

**Soil** **sample**	**Soil type^a^**	**Soil pH**	**Fertilizer** **Management**	**Number of isolates**				**Representative isolates^b^**		
				**Ca-P**	**Al-P**	**Fe-P**		**Ca-P**	**Al-P**	**Fe-P**
FS1	Andisol	6.7±0.1	Chemical	13	1	5		7	1	1
FS2	Andisol	6.6±0.0	Chemical	6	2	7		4	2	3
HK	Inceptisol	6.5±0.0	Organic	4	2	7		2	1	2
HN	Andisol	6.6±0.1	Chemical	5	2	6		3	1	1
KG1	Entisol	6.1±0.1	Organic	3	8	10		2	4	2
KG2	Entisol	6.0±0.0	Organic	13	6	9		4	1	3
NG	Entisol	6.3±0.0	Organic	8	4	11		4	3	4
SG	Entisol	6.7±0.1	Chemical	8	5	2		4	3	1
TOTAL				60	30	57		30	16	17

FS1: Fukushima site 1; FS2: Fukushima site 2; HK: Hokkaido; HN: Honmachi; KG1: Kagawa site 1; KG2: Kagawa site 2; NG: Nagano; and SG: Saga. ^a^ Based on USDA Soil Taxonomy ^b^ Screened using the phosphate solubilization index

**Table 2.** Correlation coefficients for P contents in soil samples and isolated inorganic phosphate solubilizing bacteria.

**Variables**	**Absorbable P**	**Al-Fe bound P**	**Ca bound P**	**Residual P**	**Total iPSB**	**Ca-iPSB**	**Al-iPSB**
**Al-Fe bound P**	0.91**	—					
**Ca bound P**	0.61	0.81*	—				
**Residual P**	–0.91**	–0.99***	–0.86**	—			
**Total iPSB**	–0.21	–0.22	–0.51	0.31	—		
**Ca-iPSB**	0.22	0.42	0.53	–0.42	0.00	—	
**Al-iPSB**	–0.17	–0.53	–0.74*	0.53	0.17	–0.52	—
**Fe-iPSB**	–0.12	–0.07	–0.03	0.07	–0.13	–0.75*	–0.18

Significant relationships at *** *P*<0.001 ** *P*<0.01; * *P*<0.05.

**Table 3.** Representative isolates and their classification based on genetic characterization.

**Isolate**	**Area**	**Code**	**Type of inorganic P**	**P solubilization index**	**Class**	**% Homology**	**16S rRNA**	**% Homology**	***rpoB***	**Gram Reaction^a^**	**Cellular morphology^b^**	**Colony morphology^c^**
JA1	Fukushima	FS1	Al-P	1.40	*α-proteobacteria*	99	*Pleomorphomonas sp.*	—	Not amplified (N/A)	–	coccobacilli	entire, white, convex, shiny, punctiform
JC1		FS1	Ca-P	2.90	*β-proteobacteria*	99	*Acidovorax wautersii*	96	*Acidovorax avenae*	–	coccobaccili	entire, white, convex, translucent, punctiform
JC2		FS1	Ca-P	1.50	*γ-proteobacteria*	98	*Pantoea sp.*	91	*Pantoea sp.*	–	bacilli	entire, orange, convex, translucent, punctiform
JC3		FS1	Ca-P	1.33	*γ-proteobacteria*	96	*Enterobacter sp.*	99	*Enterobacter sp.*	–	bacilli	entire, white, flat, translucent, punctiform
JC4		FS1	Ca-P	2.05	*γ-proteobacteria*	92	*Pseudomonas sp.*	95	*Pseudomonas sp.*	–	bacilli	entire, yellow, convex, translucent, convex, round
JC5		FS1	Ca-P	2.00	*β-proteobacteria*	93	*Acidovorax wautersii*	96	*Acidovorax avenae*	–	coccobaccili	entire, white, convex, translucent, punctiform
JC6		FS1	Ca-P	1.50	*γ-proteobacteria*	95	*Pseudomonas veronii*	100	*Pseudomonas veronii*	–	bacilli	entire, cream, convex, shiny, punctiform
JC7		FS1	Ca-P	1.08	*Bacilli*	98	*Priestia megaterium*	99	*Priestia megaterium*	+	bacilli	wavy, opaque, flat, matte, round
JF1		FS1	Fe-P	6.33	*β-proteobacteria*	100	*Paraburkholderia phytofirmans*	98	*Paraburkholderia sp.*	–	bacilli	entire, white, convex, translucent, punctiform
JA2		FS2	Al-P	1.25	*α-proteobacteria*	99	*Pleomorphomonas oryzae*	—	N/A	–	coccobacilli	entire, white, flat, shiny, round
JA3		FS2	Al-P	2.67	*γ-proteobacteria*	96	*Pseudomonas knackmussii*	96	*Pseudomonas knackmussii*	–	coccobacilli	entire, white, convex, translucent, punctiform
JC10		FS2	Ca-P	1.29	*γ-proteobacteria*	97	*Pseudomonas sp.*	98	*Pseudomonas mandelii*	–	bacilli	entire, yellow, convex, translucent, convex, round
JC11		FS2	Ca-P	1.42	*γ-proteobacteria*	96	*Pseudomonas sp.*	92	*Pseudomonas sp.*	–	bacilli	entire, white, translucent, convex, punctiform
JC8		FS2	Ca-P	1.28	*γ-proteobacteria*	86	*Enterobacter sp.*	100	*Enterobacter huaxiensis*	–	bacilli	entire, white, translucent, convex, punctiform
JC9		FS2	Ca-P	1.24	*γ-proteobacteria*	82	*Pseudomonas sp.*	96	*Pseudomonas sp.*	–	bacilli	entire, yellow, convex, translucent, convex, round
JF2		FS2	Fe-P	2.08	*α-proteobacteria*	99	*Methylobacterium fujisawaense*	99	*Methylobacterium fujisawaense*	–	bacilli	entire, red, shiny, convex, punctiform
JF3		FS2	Fe-P	2.00	*Actinobacteria*	98	*Mycolicibacterium fluoranthenivorans*	—	N/A	+	bacilli	entire, white, shiny, convex, punctiform
JF4		FS2	Fe-P	2.33	*β-proteobacteria*	97	*Cupriavidus sp.*	96	*Cupriavidus sp.*	–	bacilli	entire, white, convex, shiny, viscous, irregular
JA4	Hokkaido	HK	Al-P	1.00	*Actinobacteria*	94	*Rhodococcus erythropolis*	—	N/A	+	bacilli	entire, white, convex, shiny, round
JC15		HK	Ca-P	1.20	*Bacilli*	99	*Priestia megaterium*	90	*Priestia sp.*	+	bacilli	wavy, opaque, flat, matte, round
JC16		HK	Ca-P	1.29	*γ-proteobacteria*	99	*Pseudomonas viciae*	98	*Pseudomonas sp.*	–	coccobacilli	entire, white, shiny, viscous, convex, round
JF6		HK	Fe-P	2.58	*β-proteobacteria*	99	*Variovorax ginsengisoli*	94	*Variovorax ginsengisoli*	–	coccobacilli	entire, cream, convex, translucent, punctiform
JF7		HK	Fe-P	2.12	*β-proteobacteria*	99	*Variovorax ginsengisoli*	94	*Variovorax ginsengisoli*	–	coccobacilli	entire, yellow, flat, round, shiny
JA5	Honmachi	HN	Al-P	1.00	*Bacilli*	93	*Priestia sp.*	98	*Priestia megaterium*	+	bacilli	wavy, white, flat, shiny, round
JC12		HN	Ca-P	1.19	*Bacilli*	100	*Priestia megaterium*	97	*Priestia sp.*	+	bacilli	entire, opaque, shiny, flat, round
JC13		HN	Ca-P	1.11	*Bacilli*	99	*Priestia megaterium*	97	*Priestia megaterium*	+	bacilli	wavy, opaque, flat, matte, round
JC14		HN	Ca-P	1.10	*Bacilli*	100	*Priestia megaterium*	98	*Priestia megaterium*	+	bacilli	wavy, opaque, flat, matte, round
JF5		HN	Fe-P	2.00	*Actinobacteria*	95	*Mycolicibacterium fluoranthenivorans*	—	N/A	+	bacilli	entire, white, convex, translucent, punctiform
JA6	Kagawa	KG1	Al-P	6.98	*β-proteobacteria*	100	*Burkholderia diffusa*	98	*Burkholderia sp.*	–	bacilli	entire, white, convex, translucent, round
JA7		KG1	Al-P	3.60	*β-proteobacteria*	98	*Paraburkholderia atlantica*	95	*Paraburkholderia sp.*	–	coccobacilli	entire, white, convex, translucent, punctiform
JA8		KG1	Al-P	5.44	*β-proteobacteria*	94	*Paraburkholderia guartelaensis*	98	*Paraburkholderia aromaticivorans*	–	coccobacilli	entire, white, convex, translucent, round
JA9		KG1	Al-P	5.38	*β-proteobacteria*	100	*Burkholderia vietnamiensis*	99	*Burkholderia sp.*	–	coccobacilli	entire, white, convex, translucent, round
JC25		KG1	Ca-P	1.13	*Bacilli*	99	*Priestia megaterium*	98	*Priestia megaterium*	+	bacilli	wavy, flat, white, matte, round
JC26		KG1	Ca-P	1.10	*Bacilli*	99	*Priestia megaterium*	99	*Priestia megaterium*	+	bacilli	wavy, opaque, flat, matte, round
JF13		KG1	Fe-P	2.47	*β-proteobacteria*	100	*Cupriavidus sp.*	96	*Cupriavidus sp.*	–	coccobacilli	entire, white, flat, shiny, viscous, round
JF14		KG1	Fe-P	12.14	*β-proteobacteria*	99	*Burkholderia diffusa*	98	*Burkholderia sp.*	–	bacilli	entire, yellow, translucent, convex, punctiform
JA10		KG2	Al-P	6.06	*β-proteobacteria*	98	*Burkholderia sp.*	100	*Burkholderia cepacia*	–	coccobacilli	entire, neon yellow, convex, matte, brittle, punctiform
JC27		KG2	Ca-P	1.51	*γ-proteobacteria*	97	*Pseudomonas silesiensis*	96	*Pseudomonas sp.*	–	bacilli	entire, white, shiny, raised, viscous, irregular
JC28		KG2	Ca-P	1.30	*Bacilli*	99	*Priestia megaterium*	99	*Priestia sp.*	+	bacilli	entire, opaque, shiny, flat, round
JC29		KG2	Ca-P	1.34	*γ-proteobacteria*	94	*Pseudomonas viciae*	96	*Pseudomonas sp.*	–	coccobacilli	entire, white, convex, shiny, viscous, round
JC30		KG2	Ca-P	1.30	*β-proteobacteria*	99	*Trinickia soli*	92	*Trinickia sp.*	–	bacilli	entire, white, convex, translucent, viscous, round
JF15		KG2	Fe-P	9.93	*β-proteobacteria*	100	*Burkholderia diffusa*	98	*Burkholderia sp.*	–	coccobacilli	entire, white, convex, translucent, round
JF16		KG2	Fe-P	10.53	*β-proteobacteria*	98	*Trinickia sp.*	92	*Trinickia soli*	–	coccobacilli	entire, white, convex, translucent, round
JF17		KG2	Fe-P	17.00	*β-proteobacteria*	99	*Burkholderia vietnamiensis*	97	*Burkholderia cepacia*	–	coccobacilli	entire, neon yellow, convex, matte, brittle, punctiform
JA11	Nagano	NG	Al-P	1.10	*α-proteobacteria*	91	*Sphingomonas panacis*	96	*Sphingomonas panacis*	–	coccobacilli	entire, white, convex, translucent, punctiform
JA12		NG	Al-P	3.71	*β-proteobacteria*	99	*Ralstonia pickettii*	97	*Ralstonia pickettii*	–	bacilli	entire, orange, convex, shiny, round
JA13		NG	Al-P	6.17	*β-proteobacteria*	98	*Burkholderia sp.*	99	*Burkholderia sp.*	–	coccobacilli	entire, white, convex, translucent, round
JC17		NG	Ca-P	1.27	*γ-proteobacteria*	97	*Pantoea sp.*	100	*Pantoea sp.*	–	coccobacilli	entire, yellow, shiny, convex, punctiform
JC18		NG	Ca-P	1.24	*γ-proteobacteria*	95	*Pseudomonas sp.*	99	*Pseudomonas arsenicoxydans*	–	bacilli	entire, white, convex, shiny, viscous, irregular
JC19		NG	Ca-P	1.62	*γ-proteobacteria*	91	*Enterobacter sichuanensis*	97	*Enterobacter sp.*	–	coccobacilli	entire, white, convex, translucent, punctiform
JC20		NG	Ca-P	1.38	*γ-proteobacteria*	95	*Pantoea brenneri*	100	*Pantoea agglomerans*	–	bacilli	entire, yellow, translucent, convex, punctiform
JF10		NG	Fe-P	5.71	*β-proteobacteria*	99	*Paraburkholderia caribensis*	96	*Paraburkholderia sp.*	–	coccobacilli	entire, white, convex, translucent, punctiform
JF11		NG	Fe-P	5.33	*β-proteobacteria*	99	*Paraburkholderia caribensis*	96	*Paraburkholderia sp.*	–	coccobacilli	entire, white, convex, translucent, punctiform
JF8		NG	Fe-P	4.57	*γ-proteobacteria*	97	*Rhodanobacter denitrificans*	93	*Rhodanobacter denitrificans*	–	bacilli	curled, white, umbonate, irregular
JF9		NG	Fe-P	12.14	*β-proteobacteria*	100	*Burkholderia vietnamiensis*	99	*Burkholderia sp.*	–	coccobacilli	entire, white, convex, translucent, punctiform
JA14	Saga	SG	Al-P	6.04	*β-proteobacteria*	97	*Burkholderia sp.*	99	*Burkholderia sp.*	–	coccobacilli	entire, white, convex, translucent, round
JA15		SG	Al-P	4.17	*β-proteobacteria*	96	*Burkholderia sp.*	98	*Burkholderia sp.*	–	coccobacilli	entire, white, convex, translucent, punctiform
JA16		SG	Al-P	4.30	*β-proteobacteria*	100	*Burkholderia vietnamiensis*	99	*Burkholderia pyrrocinia*	–	bacilli	entire, neon yellow, convex, shiny, punctiform
JC21		SG	Ca-P	1.41	*γ-proteobacteria*	96	*Pseudomonas sp.*	96	*Pseudomonas sp.*	–	coccobacilli	entire, cream, convex, translucent, round
JC22		SG	Ca-P	1.44	*γ-proteobacteria*	94	*Pseudomonas sp.*	97	*Pseudomonas arsenicoxydans*	–	bacilli	entire, white, convex, shiny, viscous, round
JC23		SG	Ca-P	1.71	*β-proteobacteria*	99	*Burkholderia diffusa*	98	*Burkholderia sp.*	–	coccobacilli	entire, cream, convex, translucent, punctiform
JC24		SG	Ca-P	1.28	*γ-proteobacteria*	92	*Pseudomonas sp.*	97	*Pseudomonas sp.*	–	bacilli	entire, cream, convex, shiny, moist, round
JF12		SG	Fe-P	5.00	*β-proteobacteria*	99	*Trinickia soli*	92	*Trinickia sp.*	–	coccobacilli	entire, white, translucent, convex, punctiform

^a^ Gregersen’s method ^b^ Observed under an oil immersion objective ^c^ Grown on Luria-Bertani agar and observed after 24 h

**Table 4.** Summary of promising phosphate solubilizing bacteria and their growth promotion of the rice cultivar Koshihikari.

**Type of inorganic P**	**Isolate name**	**Soil sampling site**	**Bacterial species^a^**	**Shoot length (cm)**	**Root length (cm)**	**Shoot dry weight (mg)**	**Root dry weight (mg)**	**Total dry weight (mg)**
Ca-P	Control			19.0±1.7	9.2±1.0	10.4±3.9	10.7±2.1	21.1±5.8
	JR37	Reference strain	*Pseudomonas veronii*	16.0±1.3	11.2±4.8	7.9±4.2	15.6±8.0	23.5±11.0
	JC1	Fukushima S1	*Acidovorax sp.*	16.0±1.0	10.5±0.9	10.7±2.7	14.9±3.7	25.6±4.1
	JC5	Fukushima S1	*Acidovorax sp.*	15.1±1.4	14.8±2.5**	9.8±2.2	8.7±3.5	18.5±5.5
	JC11	Fukushima S2	*Pseudomonas sp.*	15.8±0.4	14.5±5.0*	5.8±0.9	10.0±2.1	15.7±2.8
Al-P	Control			16.6±1.4	11.2±0.6	8.9±1.1	9.5±2.7	18.4±3.3
	JR37	Reference strain	*Pseudomonas veronii*	14.7±2.4	11.4±1.0	7.4±2.5	3.3±1.7	10.7±2.9
	JA6	Kagawa S1	*Burkholderia sp.*	15.9±1.6	14.4±1.3^+^	11.3±1.3	11.8±2.0	23.1±1.9
	JA11	Nagano	*Sphingomonas sp.*	15.5±2.0	14.4±0.8^+^	10.1±0.6	11.6±1.6	21.8±2.2
	JA10	Kagawa S2	*Burkholderia sp.*	13.4±3.6	14.8±3.2*	10.5±4.4	9.4±4.6	19.9±9.0
	JA9	Kagawa S1	*Burkholderia sp.*	15.2±3.4	13.4±0.8	12.4±2.9	12.9±2.0	25.2±4.8
	JA12	Nagano	*Ralstonia pickettii*	14.8±0.7	13.4±0.4	12.0±1.8	12.9±6.4	24.9±7.3
Fe-P	Control			17.1±1.7	10.0±1.0	9.7±1.9	10.4±3.7	20.1±5.5
	JR37	Reference strain	*Pseudomonas veronii*	16.5±0.5	9.9±0.1	9.7±1.6	12.3±2.9	22.0±2.3
	JF3	Fukushima S2	*Mycolicibacterium sp.*	20.3±2.7	12.3±1.9	9.7±0.5	8.4±0.4	18.1±0.4
	JF6	Hokkaido	*Variovorax sp.*	17.4±3.9	14.3±1.0**	10.3±1.7	12.7±3.0	23.0±3.3
	JF5	Honmachi	*Mycolicibacterium sp.*	17.9±1.2	13.9±1.3*	10.2±0.8	11.8±1.4	22.0±1.3
	JF8	Nagano	*Rhodanobacter sp.*	16.3±3.6	11.6±1.0	12.2±8.7	10.0±2.1	22.2±10.0
	JF14	Kagawa S1	*Burkholderia sp.*	18.2±0.2	12.4±0.8	9.7±2.0	13.5±1.4	23.2±1.7
	JF15	Kagawa S2	*Burkholderia sp.*	17.4±0.4	12.2±1.1	10.7±1.6	13.2±2.5	24.0±3.9

^a^ Based on 16S rRNA and *rpoB* gene sequencing Mean±standard deviations; *n*=3. Significant increase from uninoculated with an insoluble P source (control), within each type of inorganic P (**‍ ‍*P*<0.01; * *P*<0.05; ^+^ *P*<0.1).
