# Supplementary material for: Isolation and Characterization of Phosphate Solubilizing Bacteria from Paddy Field Soils in Japan
Source: Microbes Environ. 2022 May 21;37(2):ME21085. doi: 10.1264/jsme2.ME21085 (PMC9530731; doi:10.1264/jsme2.ME21085)
Supplement: Supplementary file 1 — Supplementary Material [file 37_21085_s1.pdf]

Supplementary figures

Isolation and Characterization of Phosphate Solubilizing Bacteria from Paddy Field Soils in Japan

Jean Louise Cocson Damo[1,2], Maria Daniela Artigas Ramirez[3], Shin-ichiro Agake[1], Mannix Pedro[2], Marilyn Brown[2], Hitoshi Sekimoto[4], Tadashi Yokoyama[5], Soh Sugihara[6], Shin Okazaki[6], and Naoko Ohkama-Ohtsu[6,7*]

[1]United Graduate School of Agriculture, Tokyo University of Agriculture and Technology, Saiwaicho 3-5-8, Fuchu, Tokyo 183-8509, Japan.

[2]National Institute of Molecular Biology and Biotechnology, University of the Philippines Los Baños, Los Baños, Laguna 4031, Philippines.

[3]Iriomote Station, Tropical Biosphere Research Center, University of the Ryukyus, 870 Uehara, Yaeyama, Taketomi, Okinawa 907-1541, Japan.

[4]Faculty of Agriculture, Utsunomiya University, Utsunomiya 321-8505, Japan.

[5]Faculty of Food and Agricultural Sciences, Fukushima University, Kanayagawa 1, Fukushima, Fukushima 960-1296, Japan.

[6]Institute of Agriculture, Tokyo University of Agriculture and Technology, Saiwaicho 3-5-8, Fuchu, Tokyo 183-8505, Japan.

[7]Institute of Global Innovation Research, Tokyo University of Agriculture and Technology, Saiwaicho 3-5-8, Fuchu, Tokyo 183-8509, Japan.

***Corresponding author.**

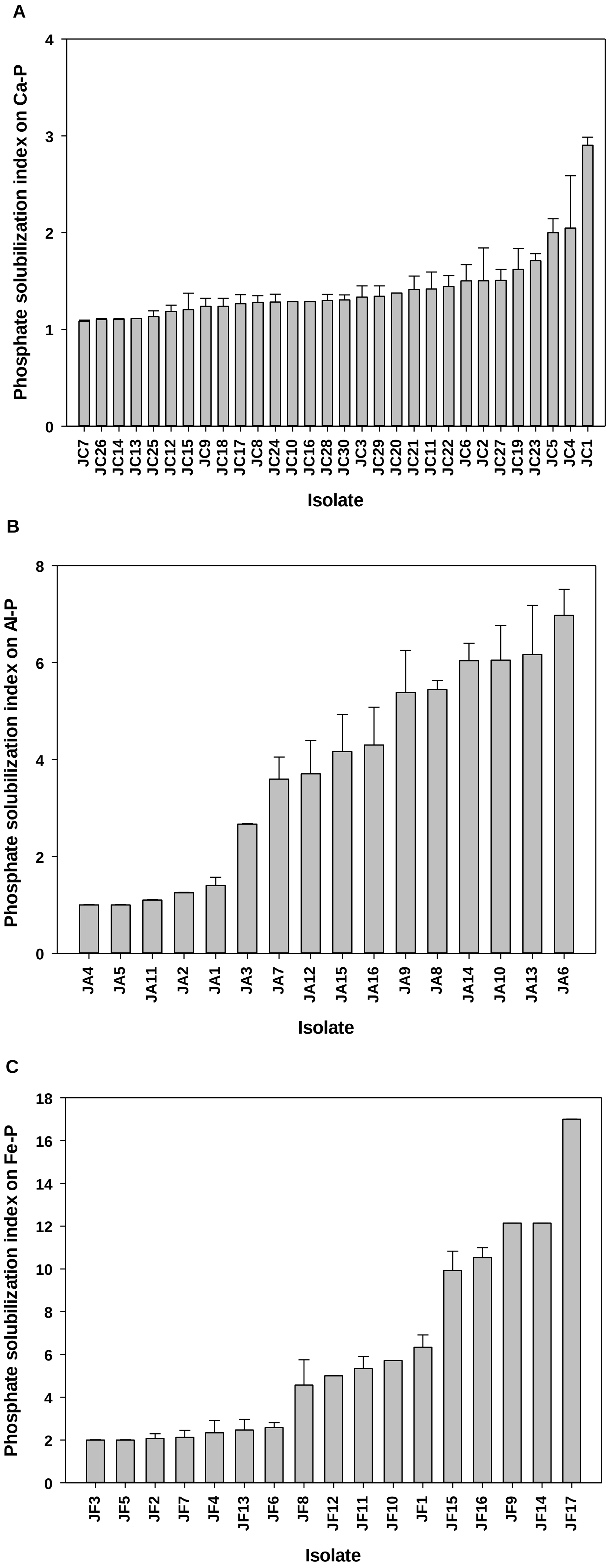

Supplementary Fig.S1. Phosphate solubilization indices for representative isolates on P growth media amended with tricalcium phosphate (Ca-P) (A), aluminum phosphate (Al-P) (B), or iron phosphate (Fe-P) (C).

Means and standard deviations (n = 3) are shown.

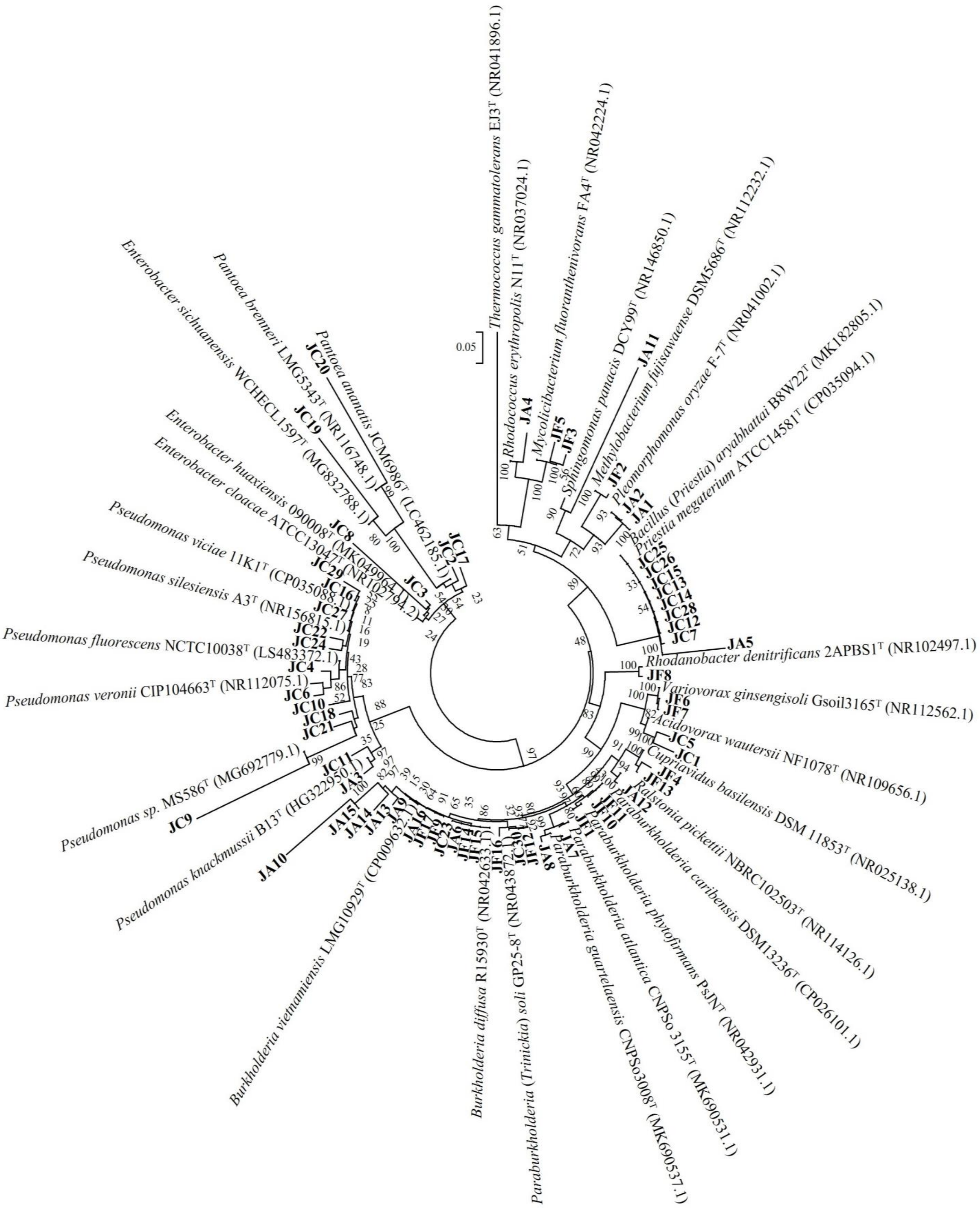

Supplementary Fig.S2. Phylogenetic tree based on 16S rRNA gene sequencing of 63 phosphate solubilizing bacteria and type strains. Numbers at the nodes indicate the level of bootstrap support (%) based on a 1,500 bp DNA fragment and neighbor-joining analysis with 1,000 replications. The scale bar indicates 0.05 changes per site.

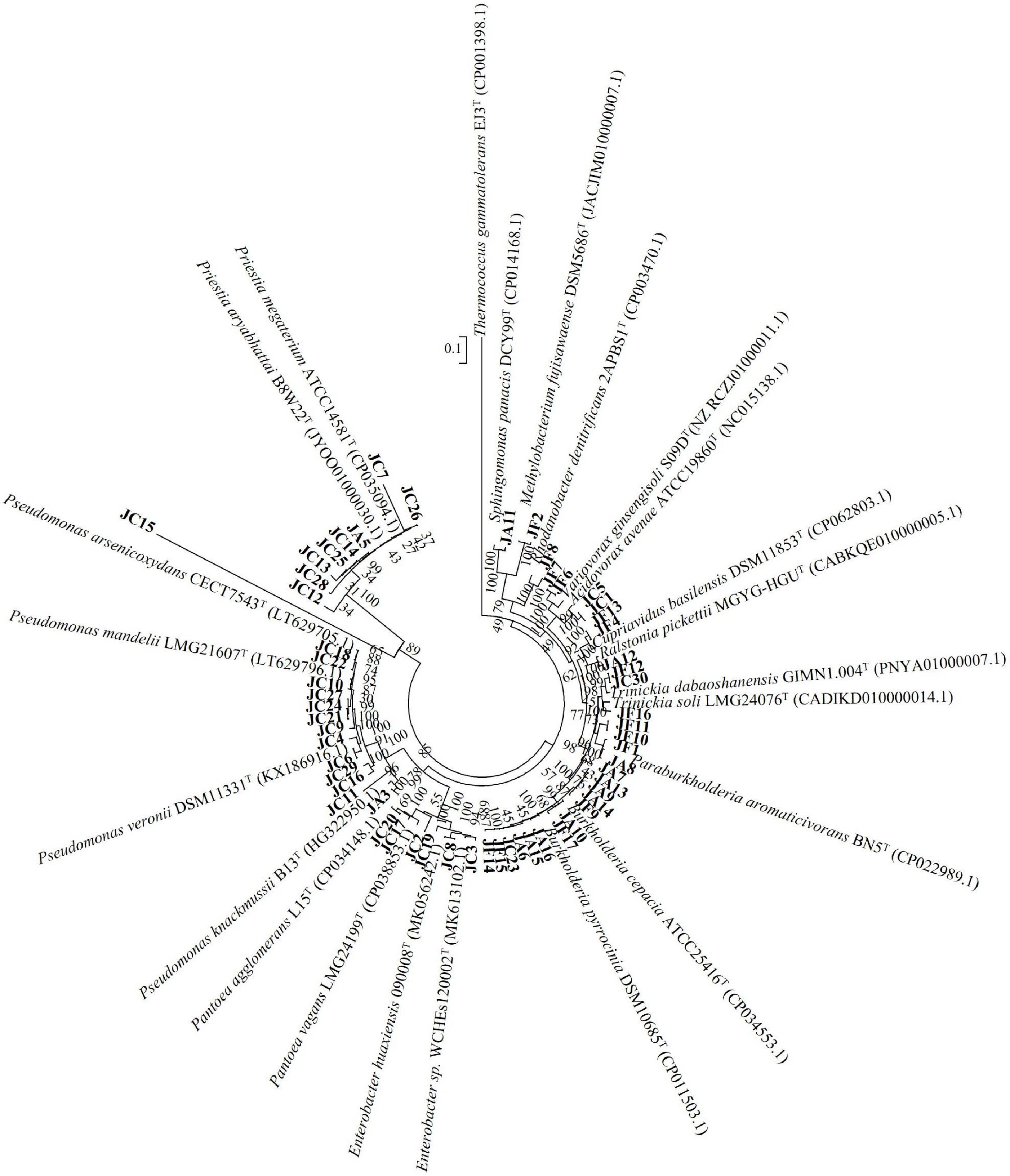

Supplementary Fig.S3. Phylogenetic tree based on *rpoB* gene sequencing showing positions of 63 isolates and type strains of species in different genera. Numbers at the nodes indicate the level of bootstrap support (%) based on a 1,500 bp DNA fragment and neighbor-joining analysis with 1,000 replications. Scale bar indicates 0.1 changes per site.

A

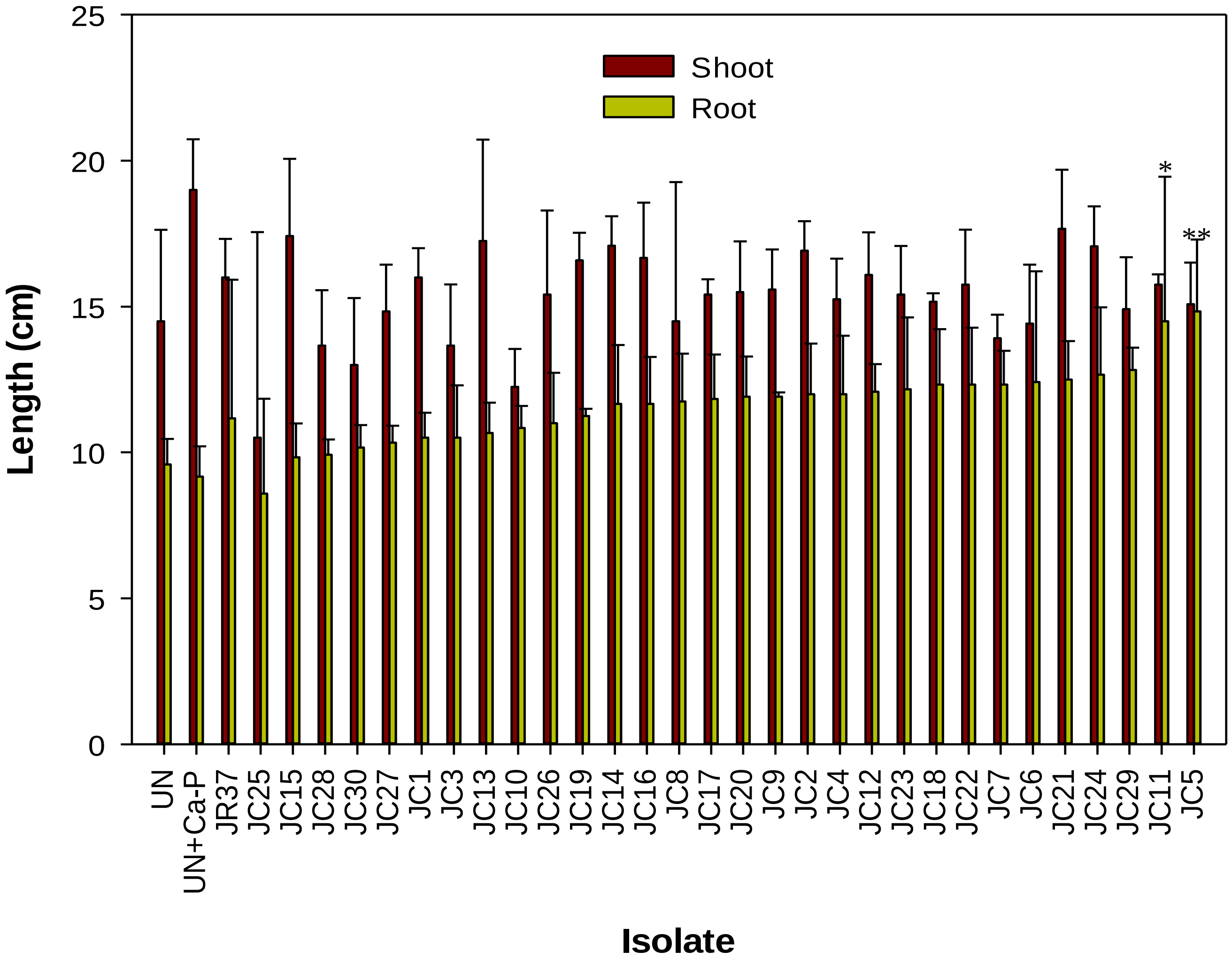

B

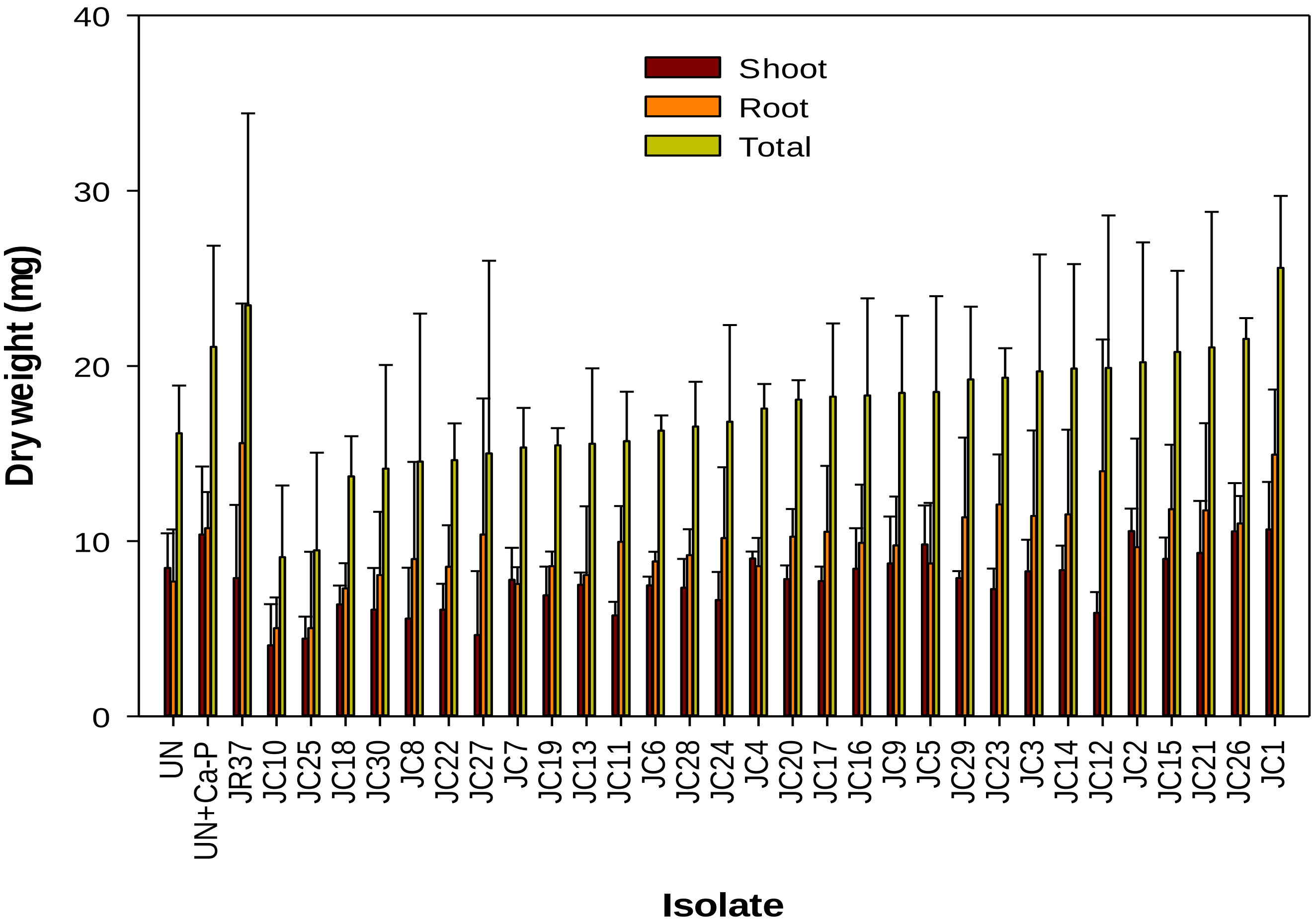

Supplementary Fig.S4. Plant growth promotion of Ca-P isolates on rice cultivar Koshihikari at 14 days after sowing.

Shoot and root length (A), Shoot, root and total dry weight (B). UN: uninoculated without tricalcium phosphate; UN+Ca-P: uninoculated with tricalcium phosphate. Significant differences between UN+Ca-P and inoculation with each isolate determined by Dunnett's test (***p*<0.01; * *p*<0.05). The error bar indicates the standard deviation of three replications.

A

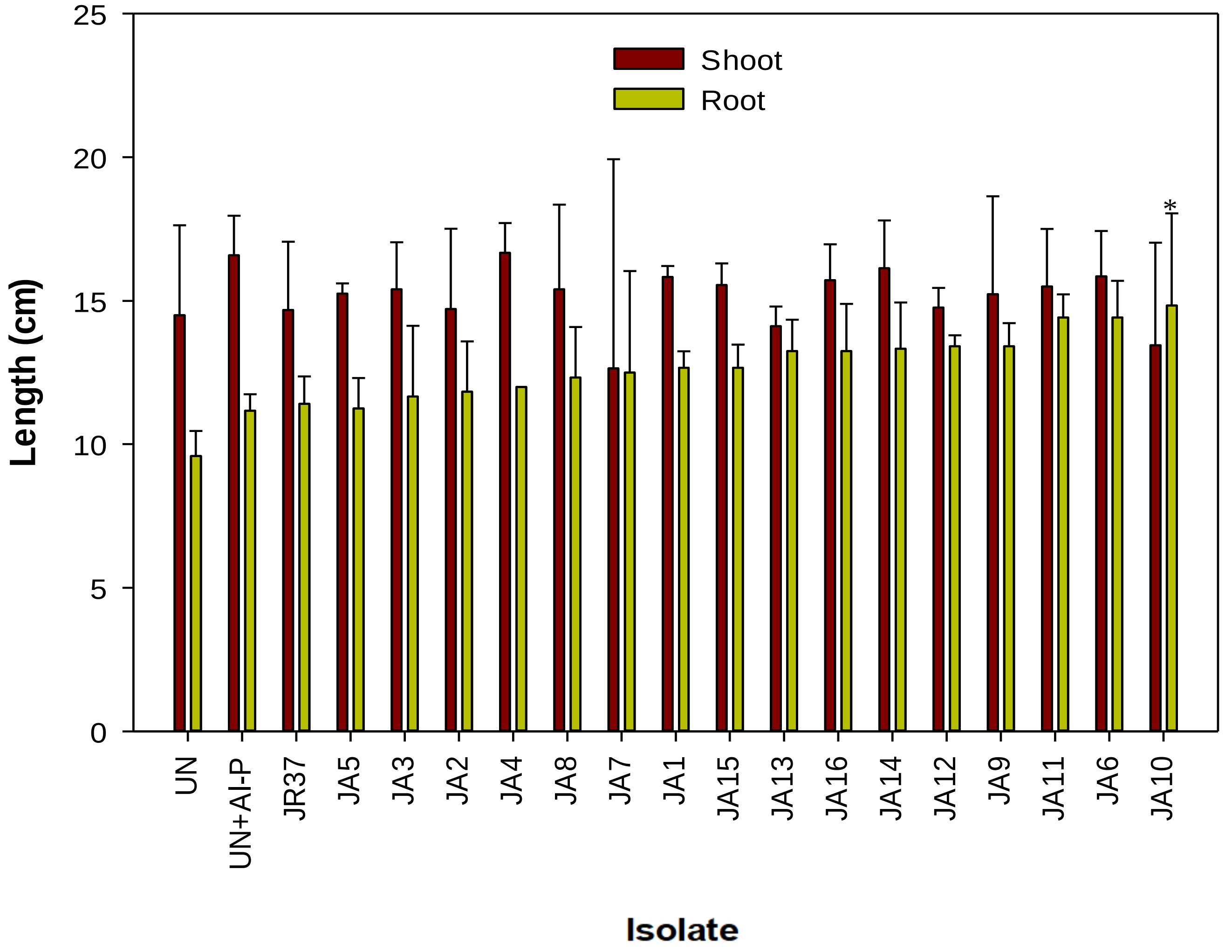

B

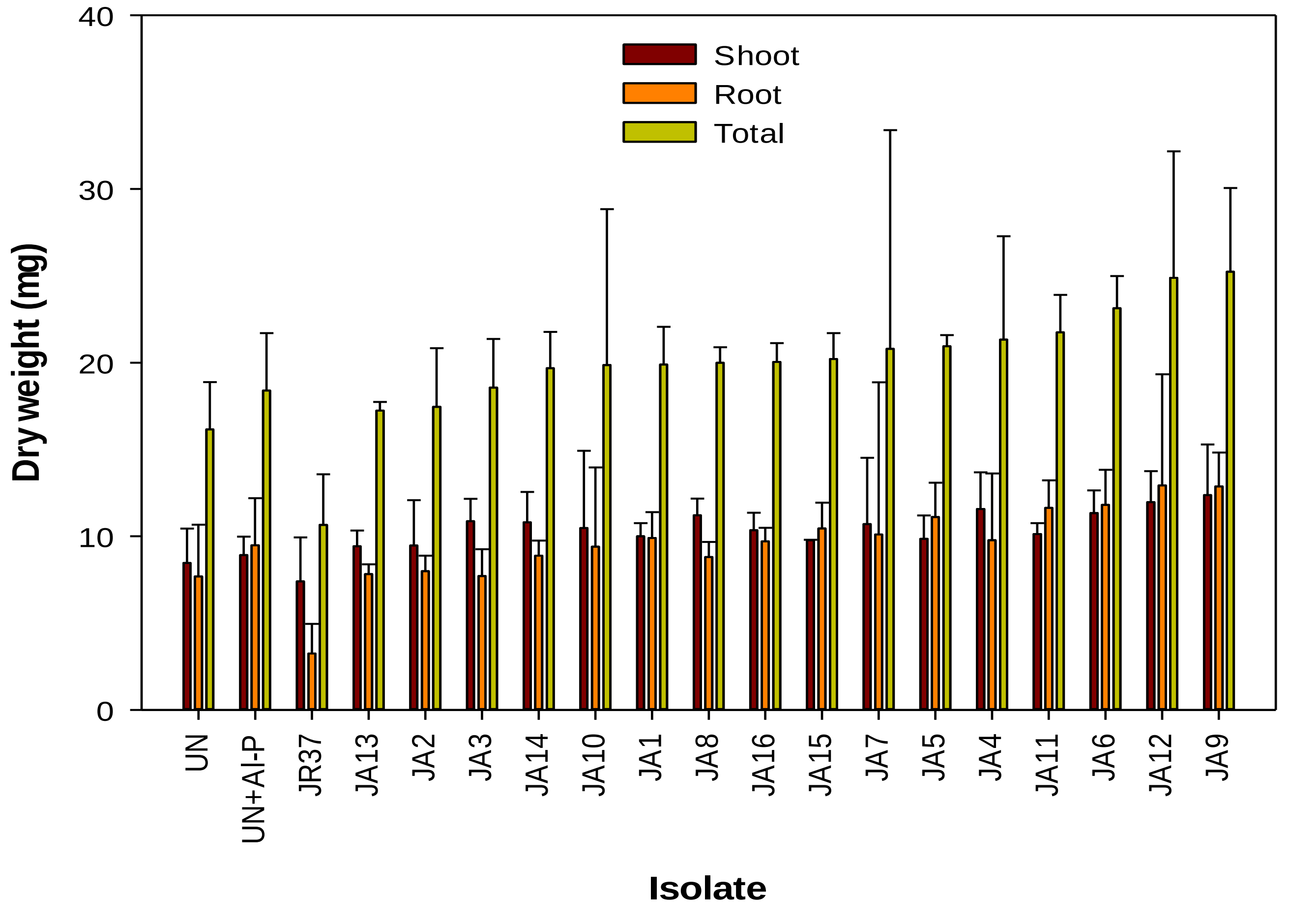

Supplementary Fig.S5. Plant growth promotion of Al-P isolates on rice cultivar Koshihikari at 14 days after sowing.

Shoot and root length (A), Shoot, root and total dry weight (B). UN: uninoculated without aluminum phosphate; UN+Al-P: uninoculated with aluminum phosphate. Significant differences between UN+Al-P and inoculation with each isolate determined by Dunnett's test (***p*<0.01; * *p*<0.05). The error bar indicates the standard deviation of three replications.

A

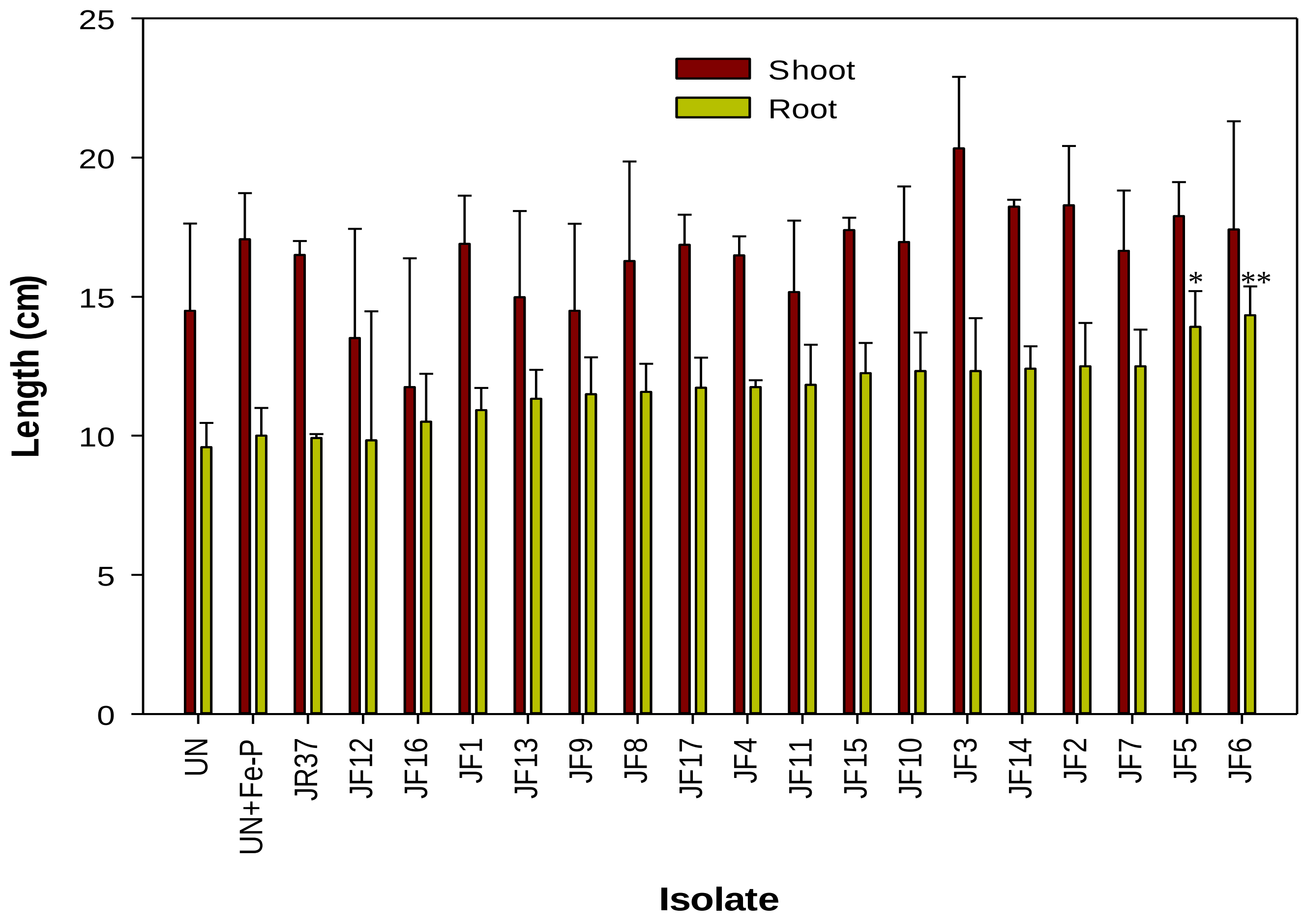

B

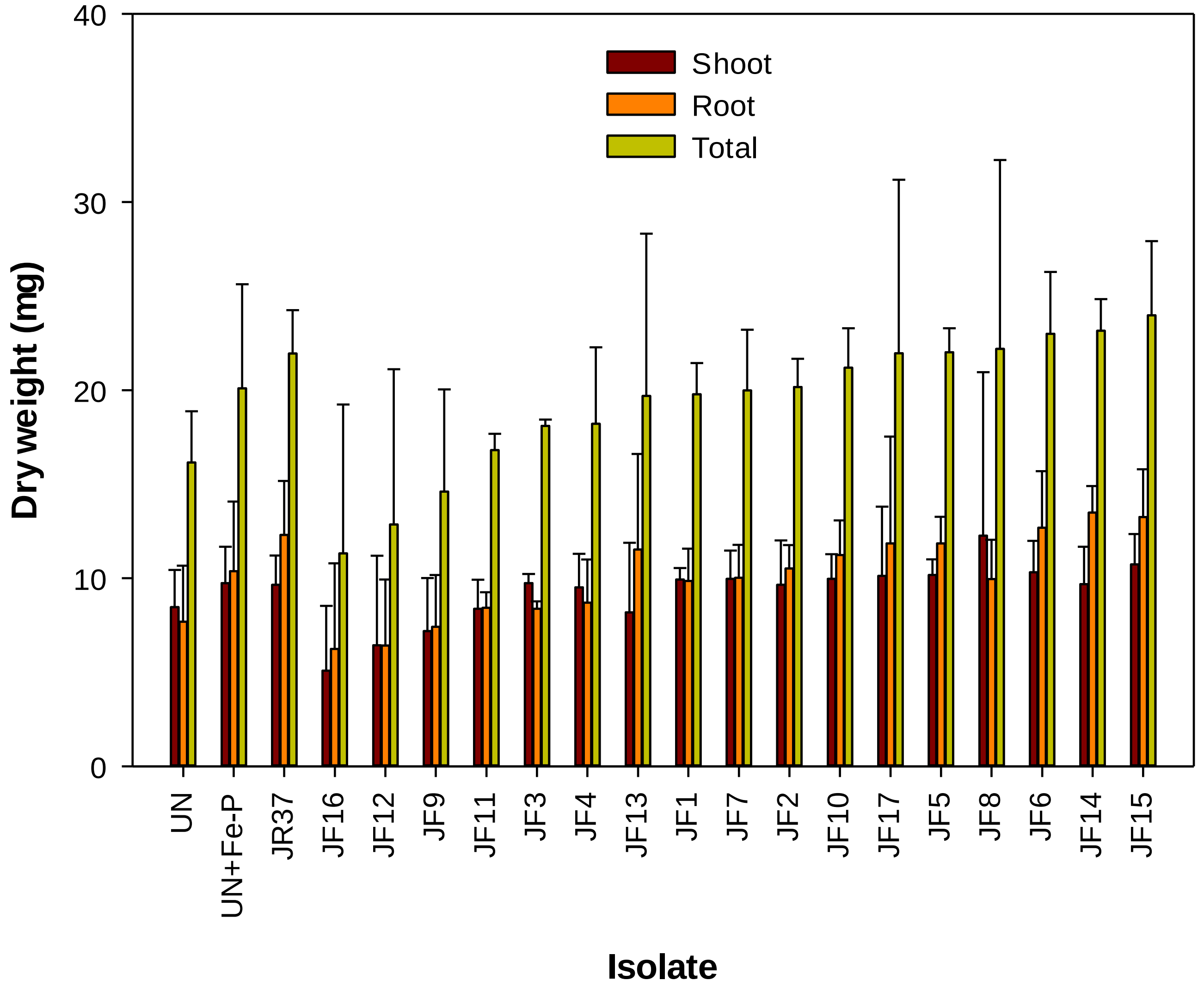

Supplementary Fig.S6. Plant growth promotion of Fe-P isolates on rice cultivar Koshihikari at 14 days after sowing.

Shoot and root length (A), Shoot, root and total dry weight (B). UN: uninoculated without iron phosphate; UN+Fe-P: uninoculated with iron phosphate. Significant differences between UN+Fe-P and inoculation with each isolate determined by Dunnett's test (***p*<0.01; * *p*<0.05). The error bar indicates the standard deviation of three replications.

Supplementary tables

Isolation and Characterization of Phosphate Solubilizing Bacteria from Paddy Field Soils in Japan

Jean Louise Cocson Damo[1,2], Maria Daniela Artigas Ramirez[3], Shin-ichiro Agake[1], Mannix Pedro[2], Marilyn Brown[2], Hitoshi Sekimoto[4], Tadashi Yokoyama[5], Soh Sugihara[6], Shin Okazaki[6], and Naoko Ohkama-Ohtsu[6,7*]

[1]United Graduate School of Agriculture, Tokyo University of Agriculture and Technology, Saiwaicho 3-5-8, Fuchu, Tokyo 183-8509, Japan.

[2]National Institute of Molecular Biology and Biotechnology, University of the Philippines Los Baños, Los Baños, Laguna 4031, Philippines.

[3]Iriomote Station, Tropical Biosphere Research Center, University of the Ryukyus, 870 Uehara, Yaeyama, Taketomi, Okinawa 907-1541, Japan.

[4]Faculty of Agriculture, Utsunomiya University, Utsunomiya 321-8505, Japan.

[5]Faculty of Food and Agricultural Sciences, Fukushima University, Kanayagawa 1, Fukushima, Fukushima 960-1296, Japan.

[6]Institute of Agriculture, Tokyo University of Agriculture and Technology, Saiwaicho 3-5-8, Fuchu, Tokyo 183-8505, Japan.

[7]Institute of Global Innovation Research, Tokyo University of Agriculture and Technology, Saiwaicho 3-5-8, Fuchu, Tokyo 183-8509, Japan.

***Corresponding author.**

Supplementary Table S1. Soil sampling sites in different regions in Japan.

**Sampling sites**	**Names of institution of the field, or places of the farmer's fields**	**Location**
Fukushima Site 1 (FS1)	Farmer's field, 37.603062^{o}N, 140.58127^{o}E	Harimichi, Nihonmatsu-shi, Fukushima Prefecture
Fukushima Site 2 (FS2)	Farmer's field, 37.603115^{o}N, 140.58098^{o}E	Harimichi, Nihonmatsu-shi, Fukushima Prefecture
Hokkaido (HK)	Farm of the Hokkaido Agricultural Research Center	Hitsujigaoka, Toyohira-ku, Sapporo City, Hokkaido Prefecture
Honmachi (HN)	Field Science Center in Tokyo University of Agriculture and Technology	Honmachi, Fuchu City, Tokyo
Kagawa Site 1 (KG1)	University Farm of Kagawa University, cv. Koshihikari was planted	Showa, Sanuki City, Kagawa Prefecture
Kagawa Site 2 (KG2)	University Farm of Kagawa University, cv. Hinohikari was planted	Showa, Sanuki City, Kagawa Prefecture
Nagano (NG)	Natural Farming International Research and Development Center	Hata, Matsumoto City, Nagano Prefecture
Saga (SG)	University Farm of Saga University Faculty of Agriculture	Shimoizumi, Kuboizumi-machi, Saga City, Saga Prefecture

Supplementary Table S2. Sequentially fractionated phosphorus in soil samples.

Soil sample	Soil type*	Soil pH	Phosphorus (P) fractions (mg/kg soil)					
			Total P	Resin P	$NaHCO_3$	NaOH	HCl	Residual P
FS1	Andisol	6.7 ± 0.1	7267 ± 696	67 ± 2 (0.9)	119 ± 4 (1.6)	509 ± 59 (7.0)	207 ± 4 (2.8)	6366 ± 700 (88)
FS2	Andisol	6.6 ± 0.0	6076 ± 235	45 ± 4 (0.7)	111 ± 8 (1.8)	420 ± 6 (6.9)	197 ± 5 (3.2)	5303 ± 232 (87)
HK	Inceptisol	6.5 ± 0.0	17288 ± 5010	75 ± 2 (0.4)	203 ± 8 (1.2)	750 ± 53 (4.3)	357 ± 34 (2.1)	15902 ± 5091 (92)
HN	Andisol	6.6 ± 0.1	21638 ± 1089	53 ± 12 (0.2)	106 ± 22 (0.5)	608 ± 25 (2.8)	462 ± 96 (2.1)	20409 ± 952 (94)
KG1	Entisol	6.1 ± 0.1	12105 ± 628	54 ± 6 (0.4)	186 ± 18 (1.5)	473 ± 49 (3.9)	119 ± 9 (1.0)	11272 ± 653 (93)
KG2	Entisol	6.0 ± 0.0	16436 ± 1777	53 ± 3 (0.3)	133 ± 13 (0.8)	550 ± 61 (3.3)	263 ± 33 (1.6)	15437 ± 1733 (94)
NG	Entisol	6.3 ± 0.0	18956 ± 696	43 ± 2 (0.2)	91 ± 2 (0.5)	635 ± 26 (3.4)	241 ± 27 (1.3)	17946 ± 728 (95)
SG	Entisol	6.7 ± 0.1	7902 ± 421	33 ± 1 (0.4)	86 ± 3 (1.1)	295 ± 16 (3.7)	136 ± 10 (1.7)	7350 ± 435 (93)

*Based on the USDA Soil Taxonomy

Numbers in the parentheses are percentages over the total P

Mean standard deviations; n=3
